# Flower color polymorphism in the peacock anemone (*Anemone pavonina*) reflects spatiotemporal variation in pollinator abundance

**DOI:** 10.1002/ajb2.70189

**Published:** 2026-04-08

**Authors:** Jonathan Heinze, Casper J. van der Kooi, Gerd Vogg, Udo Jäger, Johannes Spaethe

**Affiliations:** ^1^ Department of Behavioural Physiology and Sociobiology (Zoology II) Biocentre, University of Würzburg Würzburg 97074 Germany; ^2^ Groningen Institute for Evolutionary Life Sciences University of Groningen Groningen 9747 AG The Netherlands; ^3^ Botanical Garden University of Würzburg Würzburg 97082 Germany

**Keywords:** beetle pollination, floral signal, flower color polymorphism, flowering phenology, Glaphyridae, plant fitness, pollination, poppy guild flowers, Ranunculaceae, reproductive biology

## Abstract

**Premise:**

Flower color polymorphisms are found across angiosperms and are shaped by multiple environmental factors. We investigated *Anemone pavonina*, which displays flower color variation from red to purple along an elevational gradient on Mount Olympus, Greece. This species serves as a model for studying how elevation‐associated shifts in biotic and abiotic factors shape floral trait variation.

**Methods:**

We examined the floral biology of *A. pavonina* in the field (Greece) and in greenhouse experiments (Germany). We studied plant breeding system and flowering phenology; quantified pollinator dependence, pollen limitation, and pollinator contribution to seed set; and investigated pollinator distribution and color preferences, linking these patterns to elevation.

**Results:**

*Anemone pavonina* is protogynous, partially self‐compatible, but relies on pollinators for seed set. Both morphs experience pollen limitation, which increases with elevation in the red morph but not in the purple one. In polymorphic populations, the flowering of red morphs peaked 1–2 weeks after purple morphs. Field trapping of common flower‐visitors showed that *Pygopleurus* beetles prefer red colors, whereas bees choose non‐red colors. Beetle but not bee abundance decreased with elevation.

**Conclusions:**

*Anemone pavonina* shows clear pollinator dependence. Flower color and phenology differ among morphs, reflecting adaptation to their local environment. The purple morph is better adapted to high elevations and associated with bee pollination, whereas the red morph is tuned to beetle vision and activity patterns. These findings demonstrate how spatiotemporal pollinator dynamics drive pollinator‐mediated selection, contributing to the origin and maintenance of flower color variation.

Flower color is one of the most prominent traits of angiosperms. While interspecific color variation and evolutionary color shifts are widespread across the phylogenetic tree, intraspecific variation in flower color remains comparatively rare (Fenster et al., 2004; Narbona et al., 2018). Nevertheless, flower color polymorphism—the coexistence of two or more flower color morphs within a single species—has been documented across many angiosperm taxa (Warren and Mackenzie, 2001; Schemske and Bierzychudek, 2007; Narbona et al., 2018; Sapir et al., 2021). The underlying causes, however, are often unknown or only partially understood. Overall, prezygotic and postzygotic barriers, which affect seed production or plant fitness, are known to affect floral trait variation within and between populations (Howard, 1999; Lowry et al., 2008; Christie et al., 2022). Given that individuals of the same species within a population are expected to experience similar selective pressures (reviewed by Sapir et al., 2021), intraspecific flower color variation is particularly intriguing.

Insufficient pollen receipt or quality is a common constraint in flowering plants and reduces their reproductive success (Ashman et al., 2004; Knight et al., 2005). Consequently, selection is expected to favor floral traits that enhance pollinator attraction or effectiveness, making pollen limitation an important driver of floral diversity (Ashman et al., 2004; Knight et al., 2005; Kay and Anderson, 2025). Floral diversity is caused by both pollinator‐related factors and plant‐related factors. Pollinator‐related factors include behavior or color preference (Ortiz et al., 2015; Veiga et al., 2016; van der Kooi and Stavenga, 2019; Rodríguez‐Castañeda et al., 2024; Johnson et al., 2025), while plant‐related factors include differences in the plant mating system, for example in selfing capacity (Jiménez‐López et al., 2020), that determine pollen limitation patterns and thus influence floral traits. Further, abiotic factors such as temperature, nutrients, or precipitation may influence distributional patterns of flower color morphs (Arista et al., 2013; Grossenbacher et al., 2026; Labin et al., 2026).

Flower color is commonly determined by floral pigments, such as anthocyanins, which not only serve as visual signal for pollinators but also play critical physiological roles in plants, such as providing photoprotection or drought resistance (reviewed by Strauss and Whittall, 2006; del Valle et al., 2015; Landi et al., 2015). Reduced anthocyanin content can lower resistance to environmental stressors such as temperature extremes, drought, UV‐radiation, and challenging soil conditions. This may be caused directly through the absence of flavonoids’ antioxidant or other biochemical functions, or indirectly through pleiotropic effects (Simms and Bucher, 1996; Chalker‐Scott, 1999), and can severely affect color morph fitness (Schemske and Bierzychudek, 2001, 2007; Dick et al., 2011; Berardi et al., 2016).

Flowers of many species present colors that are attractive to potential pollinators (reviewed by van der Kooi et al., 2019), and certain flower colors are associated with specific pollinator guilds. For example, many bird‐pollinated plants display UV‐absorbing red flowers, which are attractive to red‐sensitive birds (Rodríguez‐Gironés and Santamaría, 2004; Lunau et al., 2011; Shrestha et al., 2013), whereas bee‐pollinated flowers are rarely UV‐absorbing red (Dafni et al., 1990; van der Kooi and Stavenga, 2019; León‐Osper and Narbona, 2022) because bees have poor visibility at long wavelengths (Peitsch et al., 1992). Although bird pollination is largely absent in Europe (da Silva et al., 2014), the Mediterranean basin hosts multiple plant taxa with deep red, bowl‐shaped flowers, such as species within the genera *Papaver* (Papaveraceae) and *Ranunculus*, *Adonis*, and *Anemone* (Ranunculaceae), commonly referred to as the “poppy guild” (Dafni et al., 1990; Keasar et al., 2010). These red‐flowered species are most likely pollinated by flower‐visiting glaphyrid beetles (Dafni et al., 1990), which, similar to many butterflies (Stavenga and Arikawa, 2006), have spectral sensitivity that extends well into the red part of the light spectrum (Martínez‐Harms et al., 2012; Belušič et al., 2025). However, even within the poppy guild, some species exhibit remarkable intraspecific flower color variation. For instance, in *Anemone coronaria*, populations display a range of color morphs, including red, blue, white, and purple (Horovitz et al., 1975), likely maintained by a combination of pollinator‐mediated selection and climatic and edaphic factors (Horovitz, 1976; Keasar et al., 2010; Labin et al., 2026).

Growing along the slopes of Mount Olympus in Greece, *Anemone pavonina* Lam. (Ranunculaceae) is a promising study system for investigating the underlying processes that cause and maintain flower color polymorphism. Given that flower color variation in *A. pavonina* occurs across spatial scales of <1 km, it is unlikely that large‐scale environmental patterns, such as day length or precipitation, cause this color variation. Flower color ranges from red (500–1000 m a.s.l.) to purple (>1200 m a.s.l.), including a narrow transition zone of polymorphic populations (1000–1200 m a.s.l.) with red, purple, and intermediate colors (Figure 1; Streinzer et al., 2019). The factors that cause this sharp color change along the elevational gradient are unknown, although color morph abundance seems to be linked to dominant flower visitors. We hypothesized that the abundance of *Pygopleurus* beetles, the most commonly observed flower‐visitor of red “poppy‐guild” flowers (Dafni et al., 1990; Keasar et al., 2010), declines with elevation. Bees, which also visit *Anemone* species when foraging for pollen, may become more important at high elevations, replace beetles as pollinators, and select for alternative flower colors. In addition to pollinator‐mediated selection, abiotic factors—particularly temperature, which varies along the elevational gradient—may influence color morph distribution through morph‐specific traits associated with flower color, which in turn can affect stress tolerance and reproductive fitness (Strauss and Whittall, 2006).

**Figure 1:**
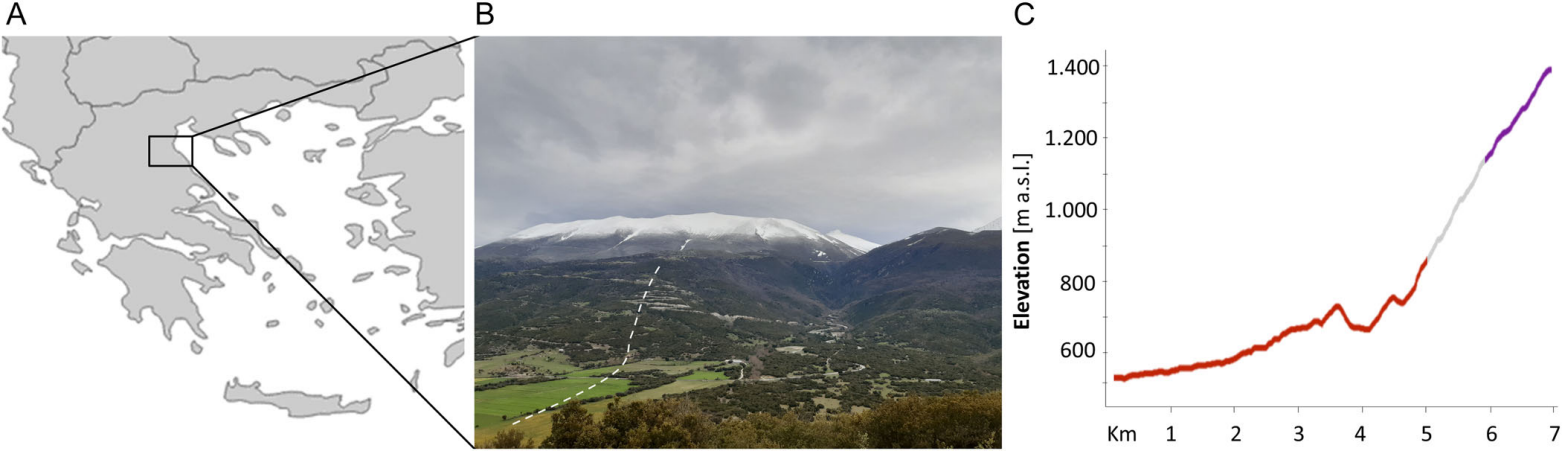
(A) Mount Olympus is located in the northern part of Greece, within the region of Thessaly. (B) Southward‐facing slopes of Mount Olympus. (**C**) Elevation graph corresponding to dashed line in B; colors indicate population type and flower color of *Anemone pavonina* (red = monomorphic red population, gray = polymorphic population [red and purple flowers], purple = monomorphic purple population).

We assessed the flowering ecology and biotic and abiotic factors that influence flower color polymorphism in *A. pavonina*, laying the foundation for further research on plant color evolution in poppy guild flowers. Specifically, we (1) examined temporal patterns of female and male phase and (2) examined breeding system (cross‐pollination, geitonogamous, and self‐pollination), auto‐fertility (AFI), and self‐compatibility (SC). We (3) assessed inter‐morph compatibility and phenology to investigate prezygotic barriers between the morphs, (4) assessed elevational and seasonal effects on plant fitness, and (5) recorded soil temperature throughout the year. To elucidate the role of pollinators, we (6) further quantified pollinator dependency (PD), pollen limitation (PL), and pollinator contribution (PC) and (7) determined distribution and color preferences of pollinators along the elevational gradient. This study offers insights into the emergence of intraspecific flower color variation and helps us understand how habitat characteristics, changing abiotic factors, and pollinator assemblage shape and maintain flower color polymorphism.

## MATERIALS AND METHODS

### Study species and region

**Figure 2:**
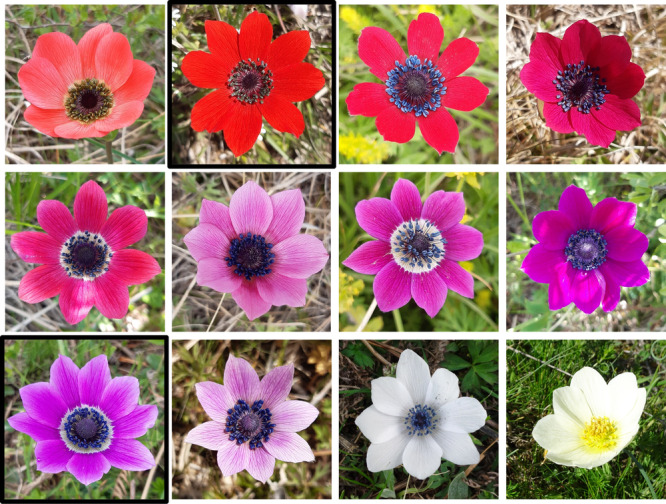
Examples of *Anemone pavonina* color morphs and an albinotic individual (bottom right). Highlighted red and purple flowers occur in monomorphic populations and are the dominating colors (~75%) in polymorphic populations. For morph quantity at each study site, see Appendix S1: Table S1.

*Anemone pavonina* (peacock anemone, Ranunculaceae) is a herbaceous perennial with an overwintering corm. It is distributed within the northern range of the Mediterranean region, mainly the central and eastern parts (Madahar, 1967). Its only reward to flower‐visiting insects is pollen (Shmida, 1981; Dafni et al., 1990). Previous work by Streinzer et al. (2019) reported elevation‐dependent color polymorphism (Figure 1) and suggested that the pure red (UV‐absorbing) morph is probably pollinated by beetles, whereas purple flowers—which reflect light in the UV‐blue range—are visited by bees. Given that UV‐absorbing red flowers are not conspicuous to bees (Peitsch et al., 1992; León‐Osper and Narbona, 2022), bees might select for purple colors at high‐elevation populations where red‐sensitive beetles become rare. In addition to red and purple colors, which comprise ~75% of all individuals in polymorphic populations, intermediate and (rarely) white colors occur (Figure 2).

To examine the flowering ecology of *A. pavonina*, we performed greenhouse and field experiments over three consecutive years. All field experiments took place along the southward‐facing slopes of Mount Olympus (Figure 1B), where *A. pavonina* is commonly found flowering from March to May. We chose 11 (2022, 2024) and 17 populations (2023) equally distributed along an elevational gradient from 330 to 1435 m a.s.l. (Figure 1C; Appendix S1: Table S1). The greenhouse experiments were performed at the Botanical Garden of the University of Würzburg, using *A. pavonina* plants that were collected on Mount Olympus in 2012. No supplemental illumination was used. The photoperiod corresponded to the natural seasonal variation in day length in Würzburg, Germany. The plants were grown in a mixed substrate containing soil, sand, and gravel, watered every 2–3 weeks, and kept in a frost‐free greenhouse during winter (minimum temperature 2.3°C). The average annual temperature was 11.5°C. During summer, the greenhouse was ventilated (open sides or with a fan), which provided constant air circulation.

### Dichogamy and plant breeding system

To characterize the duration of male and female phases, we observed *A. pavonina* flowers at the Botanical Garden of Würzburg in 2023 for 14 days after opening. Prior to the experiments, all flower buds (and later all flowers) were covered by mesh bags (12 × 15 cm, mesh width about 0.55 × 0.35 mm) to prevent insect pollination. Although flower‐visiting thrips might be able to enter the mesh bags (Pop et al., 2025), we never observed thrips on our flowers, concluding that their importance as pollinators is negligible. We examined 6 flowers per individual and 11 individuals per color (66 flowers per color in total). We checked the flowers every day for pistil appearance and dehiscence of anthers. We used the day of first pollen release as indicator for the beginning of the male phase. To investigate duration and stigma‐receptivity of the female phase, six groups (11 individuals each; one flower per individual) of red and purple individuals were used. Each group was hand pollinated on different days (from day 1 to day 6 after anthesis) with pollen of different individuals of the same color morph (cross‐pollination). For hand pollination, we used a small paintbrush (round brush, synthetic bristles, size 10), which was cleaned after each pollination event with 70% EtOH. The infructescences were collected after 2–3 weeks. We counted the number of carpels per infructescence and checked for presence of seeds (in *A. pavonina*, each carpel contains a single ovule, producing one seed). Finally, we calculated the relative seed set per individual as the number of seeds divided by the number of carpels.

To quantify self‐compatibility and capacity for autonomous self‐fertilization, we added a second and third treatment to our setup (11 individuals per color) and hand pollinated newly emerged flowers (max. 1–2 days after opening) of both colors with pollen of another flower of the same individual (geitonogamous pollination) and bagged unmanipulated flowers (autonomous self‐pollination). We calculated the self‐compatibility index (SCI; Becerra and Lloyd, 1992; Lloyd and Schoen, 1992) as the ratio of seed set after geitonogamous pollination divided by seed set after cross‐pollination, and the auto‐fertility index (AFI; Lloyd, 1965; Lloyd and Schoen, 1992) by dividing seed set after autonomous self‐pollination by that of cross‐pollination.

### Quantification of inter‐morph compatibility

Inter‐morph compatibility of red and purple flowers was investigated in 2023 in a field experiment in a color polymorphic population in Greece. We used four different hand‐pollination treatments (for procedure, see above) with 15 individuals each: (1) red flowers pollinated by pollen of red flowers from different individuals; (2) red flowers pollinated by pollen of purple flowers; (3) purple flowers pollinated by pollen of red flowers; and (4) purple flowers pollinated by pollen of purple flowers from different individuals.

### Flowering phenology, pollination dynamics, and elevational and seasonal effects

The experiments in 2022 were performed in four monomorphic red, three monomorphic purple, and four polymorphic populations (for morph proportions per population, see Appendix S1: Table S1). In 2023, we used eight monomorphic red, four monomorphic purple, and five polymorphic populations. To elucidate seasonal effects on the relative proportion of color morphs, we conducted transect walks (in 2023) once a week on a permanently marked 10 m transect on 11 sites and counted all red, purple, and mixed‐color flowers within 0.5 m left and right of the transect. We estimated the flowering peak for the red and purple morphs by fitting a fifth‐degree polynomial to the six data points obtained from the transect walks.

Further, we measured soil temperature at the same populations along the elevational gradient between November 2022 and April 2023. This covers the period when *A. pavonina* starts to grow leaves in response to the beginning of regular rainfall after the dry summer. Temperature during this time is assumed to be important for flowering onset in the following spring. We used two data loggers (Thermochron iButton DS1921G‐F5#, Maxim Integrated, San Jose, California, USA) with protective silicone cases (SL50‐ACC06‐PK1) per site. The data loggers were buried ~4 cm deep in the ground, which is the average depth of the *Anemone* corms. For average temperature, we calculated the mean of both data loggers per site.

To quantify pollen limitation, variation in pollinator contribution to seed set, and fecundity under natural pollination in relation to elevation, we used an extended pollinator exclusion experiment in 2022, with three different treatments. (1) To exclude pollinators but allow wind and autonomous self‐pollination, we covered buds that were about to open with small green mesh bags supported by a metal stick (12 × 15 cm, mesh width about 0.55 × 0.35 mm). (2) We kept a second group unmanipulated to allow open pollination. (3) In the third group, we used a standard pollen supplementation treatment and combined open pollination and hand pollination (for procedure, see above) with pollen from two or three flowers of the same color morph and the same population. All plants were labeled individually. Altogether, 405 flowers were marked: 10 individuals per color morph in each treatment per population (one red and one polymorphic population comprised only five labeled individuals per treatment). According to a randomized block design, we arranged them in 10 triplets per site, each contained one individual of each treatment. After one week, we also covered all individuals from the open‐pollination and hand‐pollination treatments with mesh bags to protect the infructescence and to provide similar conditions for seed development among all treatments.

To enable a more detailed quantification of relative seed set along the elevational gradient and during flowering season, we focused only on open pollination in 2023 and marked individual flowers at three different time points, referred to hereafter as early‐season (7–20 April), mid‐season (21 April–3 May), and late‐season (4–14 May), based on observations in 2022. During early‐ and late‐season, we sampled 11 populations and 10 individuals per population (one polymorphic population comprised only five labeled individuals per color in early‐season). During mid‐season, 17 sites, and 12 individuals per color, per site were used, resulting in a total of 554 marked plants. We covered all individuals with mesh bags after one week. After 2–3 wk, we collected all infructescences and calculated the relative seed set per individual (see above).

The data from 2022 were used to calculate pollinator dependence (PD) as 1 − (relative seed set pollinator exclusion/relative seed set open pollination), pollen limitation (PL) as 1 − (relative seed set open pollination/relative seed set additional pollen supply), and pollinator contribution (PC) as (relative seed set open pollination − relative seed set pollinator exclusion)/relative seed set open pollination (Ashman et al., 2004; Knight et al., 2005; Rodger et al., 2021).

### Pollinator color preference and distribution

To quantify pollinator abundance across the elevation of the study sites and experimentally determine pollinator color preference, we monitored insect catches (Appendix S1: Table S2) in purple, red, blue, and white cross‐vane traps (7 cm diameter, 12.5 cm total height; see Appendix S1: Figure S5) over three consecutive years (2022–24). Traps were mounted at ~30 cm above the ground and placed within 17 *Anemone* populations along the elevational gradient (330–1435 m a.s.l.). They were filled with water and some odorless detergent (Ecover Zero, Ecover Deutschland GmbH, Hamburg, Germany) to reduce surface tension. At each population, we placed 12 traps (three sets of all four colors) for 6–8 h per day, over 3–5 days per season. Trap content was analyzed both in the field and in the lab. All collected insects were identified to the order level. Based on Streinzer et al. (2019) and personal observations, bees and beetles (*Pygopleurus*) were considered the most important pollinators, and our analysis thus focused on these groups.

### Statistics

Statistical analyses were performed in R version 4.2.1 2022‐06‐23, including the additional packages “rstatix” (Kassambara, 2023), “MASS” (Venables and Ripley, 2002), “agricolae” (de Mendiburu, 2021), “car” (Fox and Weisberg, 2018), “lme4” (Bates et al., 2015), “lmerTest” (Kuznetsova et al., 2017), “glmmTMB” (Brooks et al., 2017), and “emmeans” (Lenth, 2023). For a detailed description of the statistics and a table including all used models, see Appendix S2: Table S1.

To analyze how number of carpels or day of pollen release depends on flower color, we used a Wilcoxon test because of non‐normally distributed residuals and variance inhomogeneity in our data. We used a Kruskal‐Wallis test for comparison of three or more groups (i.e., day of pollination, pollination type, and inter‐color compatibility), followed by a Dunn post hoc test to compare the groups pairwise and Bonferroni correction to account for multiple testing.

For bee and beetle (*Pygopleurus*) catches in pan traps, we calculated pollinator frequency (number of individuals per hour, standardized for the number of traps per session). We applied generalized linear models for a linear regression to analyze the relationships between temperature and elevation, flowering peak and elevation, and pollinator frequency and elevation and to further investigate the effect of elevation on PL, PD, and PC. General linear mixed models were applied for our pollinator exclusion experiments and to study seasonal or elevational effects on relative seed set and number of carpels. For the pollinator exclusion experiment, we used treatment, flower color, and population type as independent variables; for the seasonal effect experiment, the independent variables were seasonal phase, flower color, and population; to evaluate effects on relative seed set and number of carpels, we used flower color, population, and year as independent variables. We used a linear mixed‐effects model to investigate differences in flowering peak between the two color morphs, and differences in color preference of beetles and bees between monomorphic and polymorphic populations. We included study site as random factor in all mixed models to account for natural variability between sites (e.g., due to site‐specific differences in seed set attributable to varying biotic and abiotic factors). For pairwise comparison of the groups, we performed a post hoc test (“emmeans” function from the package “emmeans”). Results are presented as means ± SE.

## RESULTS

### Dichogamy and plant breeding system

**Figure 3:**
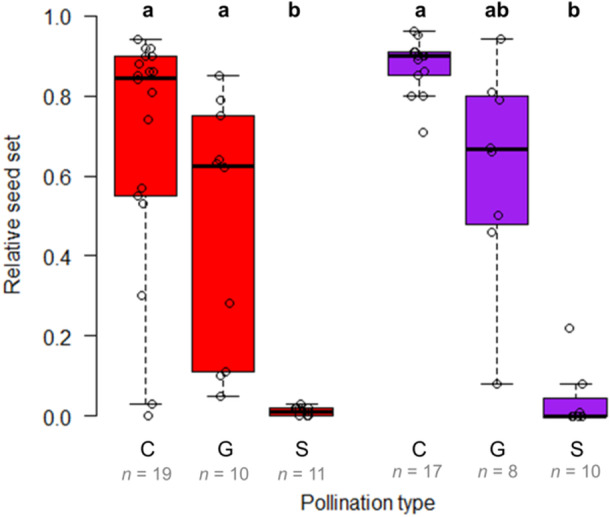
Relative seed set dependent on different pollination scenarios in hand‐pollinated *Anemone pavonina* flowers of the same color (C = cross‐pollination, G = geitonogamous pollination) and autonomous self‐pollination (S) as control. Flower color is indicated by plot color.

The temporal development of anthers and pistils showed that *A. pavonina* is protogynous (Appendix S1: Figure S1). At the beginning of the female phase, the pistils bend outward in all directions. During the transition to the male phase, the pistils bend, slightly twisted, back toward the center. The anthers dehisced gradually over several days. On average, for both color morphs, the anthers started to dehisce 4 days after the flower had opened (*P* = 0.169, *W* = 1331.5; Appendix S1: Figure S2A). We observed a mean of 274 ± 6 carpels per flower, independent of color (*P* = 0.485, *W* = 2758.5; Appendix S1: Figure S2B). Surprisingly, female receptivity decreased over time in purple individuals (*P* < 0.001, df = 5, *χ*² = 24.23; Appendix S1: Figure S3; Appendix S2: Table S2), but not in red individuals (*P* = 0.124, df = 5, *χ*² = 8.64; Appendix S2: Table S3). Relative seed set of purple flowers that were pollinated within the first 2 days after anthesis was ~5× greater than that of flowers that were pollinated on day 5 or 6. A similar but less pronounced pattern was observed in red individuals, although greater variation renders these differences not statistically significant. In some rare cases (two individuals, i.e., 2% of all flowers), there was an overlap between the female and male phases, which would in principle allow autonomous self‐pollination. Autonomous self‐pollination resulted in a low average seed set of 3 ± 1%, whereas seed set through geitonogamous pollination was 55 ± 7%, and cross‐pollination yielded 78 ± 4% (red: *P* < 0.001, df = 2, *χ*² = 23.23; purple: *P* < 0.001, df = 2, *χ*² = 18.97; Figure 3; Appendix S2: Table S4). However, the difference between cross‐pollination and geitonogamous pollination was not statistically significant. The auto‐fertility index was 0.02 for the red morph and 0.05 for the purple morph. Both morphs showed limited self‐compatibility with an SCI of 0.7.

**Figure 4:**
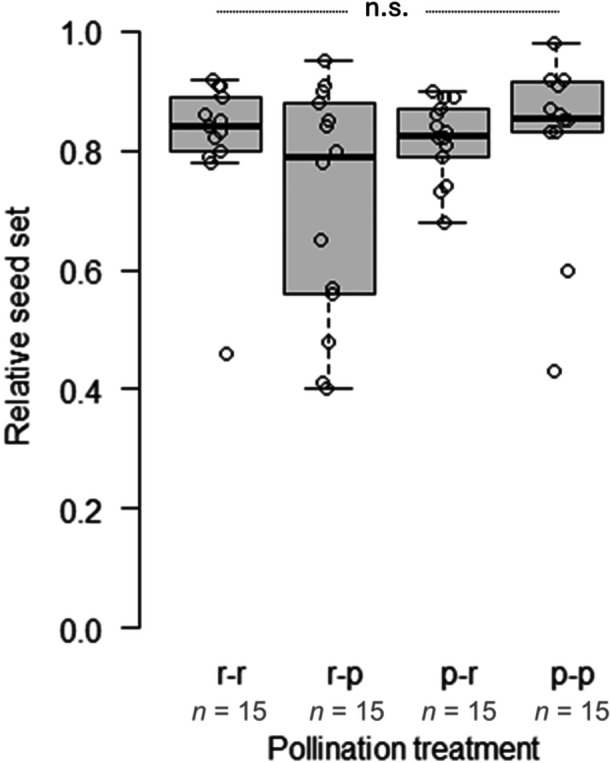
Relative seed set between the four possible inter‐morph crossing scenarios in *Anemone pavonina* (r‐r = red flower pollinated by pollen of a red flower, r‐p = red flower pollinated by pollen of a purple flower, p‐r = purple flower pollinated by pollen of a red flower, p‐p = purple flower pollinated by pollen of a purple flower). All flowers were pollinated by hand with pollen of two or three other flowers. n.s. = not significant.

Relative seed set was independent of the flower color of pollen donor or recipient (Figure 4), indicating no evidence of incompatibility or negative effects from inter‐color crossing of red and purple color morphs (*P* = 0.284, df = 3, *χ*² = 3.79; Appendix S2: Table S5).

### Flowering phenology, pollination dynamics, and elevational and seasonal effects

**Figure 5:**
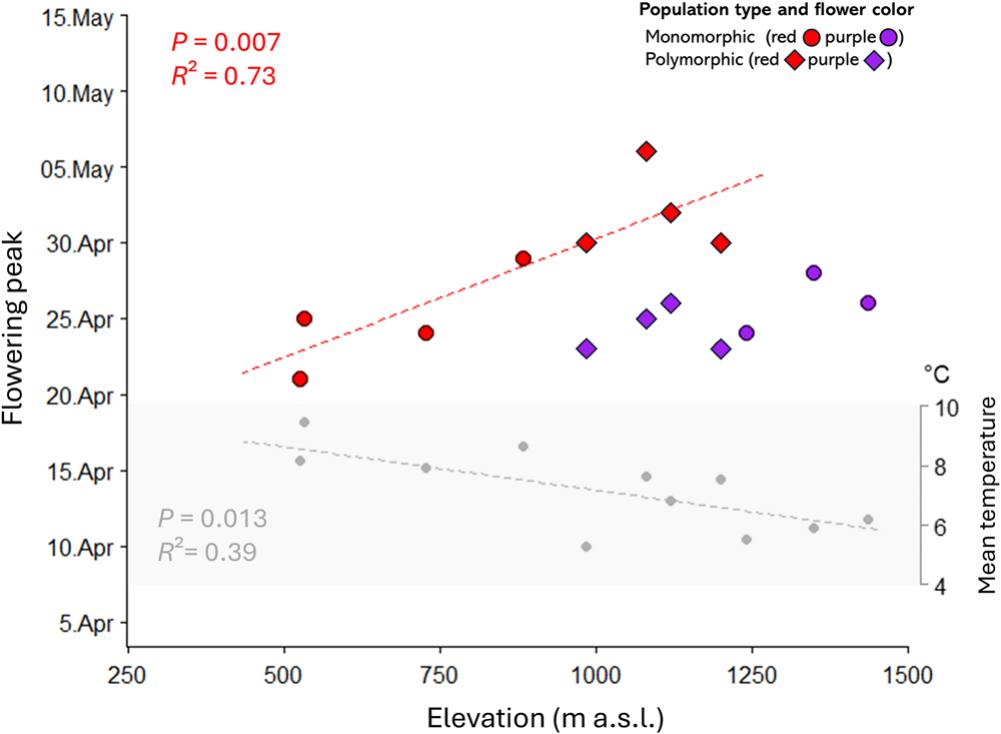
Flowering peak (date) of red and purple *Anemone pavonina* flowers and mean temperature (gray dots) during the growing season (November–April) in 2023 of different populations plotted against elevation. Monomorphic populations (●): red (500–800 m a.s.l.), purple (1200–1500 m a.s.l.). Polymorphic populations (♦): 1000–1200 m a.s.l. Dashed line indicates significant relationship.

The transect walks in 2023 (Appendix S1: Table S1) showed that most individuals in polymorphic populations were either purple (50 ± 10%) or red (27 ± 11%), depending on elevation. The proportion of intermediate colors was less variable (23 ± 2%). The transect walks further revealed a shift in phenology along the elevational gradient depending on flower color (*P* < 0.001; Appendix S2: Table S9). In the red morph, flowering peaked later in the season with increasing elevation (*R*² = 0.73, df = 6, *P* = 0.007; Figure 5). However, we did not find this effect in the purple morph (*R*² = 0.00, df = 65, *P* = 0.172). That is, the flowering peak of purple individuals in monomorphic and polymorphic populations (985–1435 m a.s.l.) or red individuals of monomorphic populations (500–882 m a.s.l.) was around 25 April (2023), whereas the peak of red individuals in polymorphic populations (985–1200 m a.s.l.) was shifted by 1.5–2.0 weeks. This shows that purple individuals started flowering earlier than red ones under the same conditions but at the same time at much higher elevations and lower temperature.

The average temperature during the growing period (November 2022 to end of April 2023) was 8.7°C in red, 7.0°C in polymorphic, and 6.1°C in purple populations (Figure 5). Overall, temperature decreased by 0.32°C per 100 m increase in elevation (*R*² = 0.39, df = 13, *P* = 0.013).

**Figure 6:**
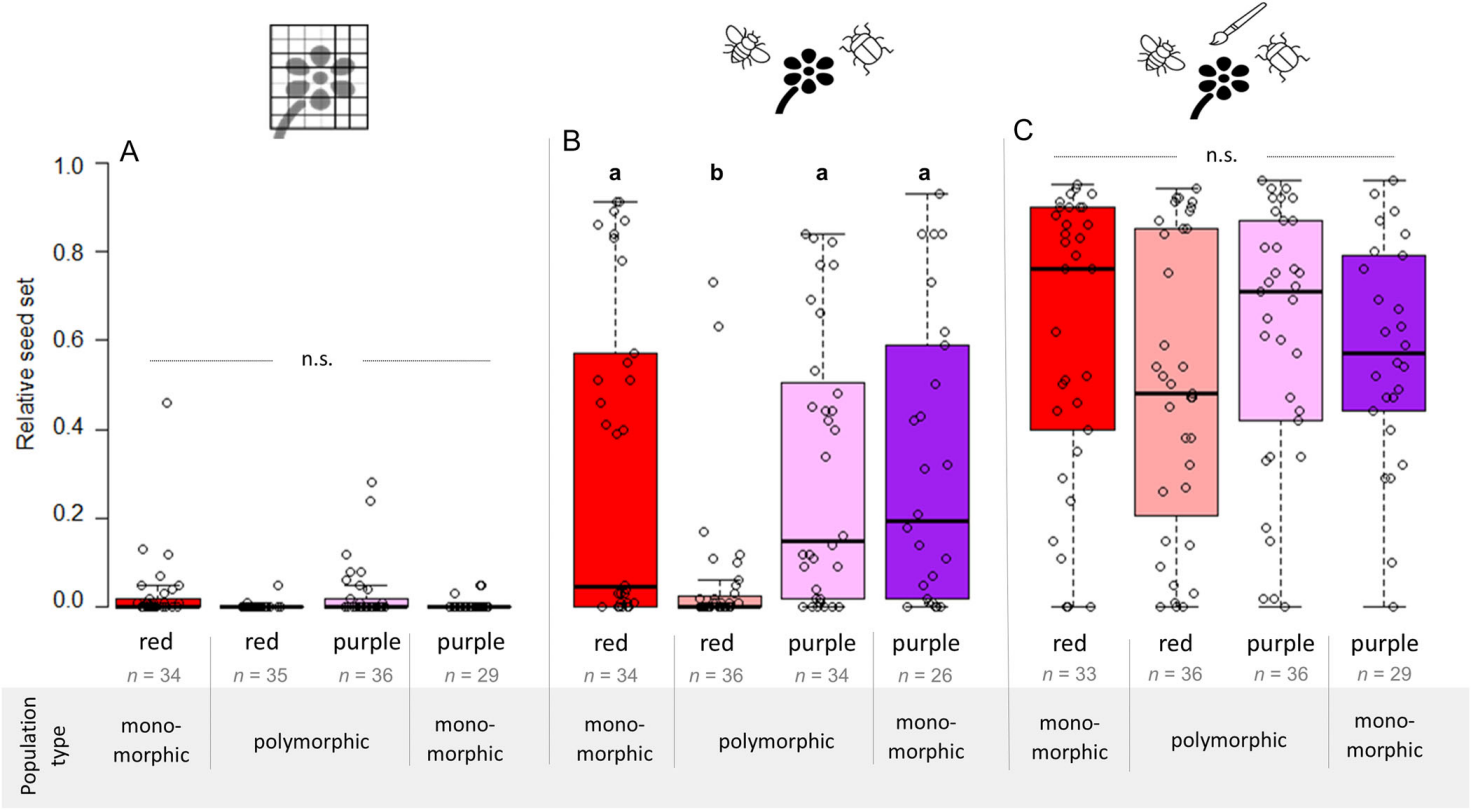
Relative seed set of *Anemone pavonina* for three different pollination treatments. (A) Closed buds were covered with a mesh bag to exclude pollinators but allow wind‐ and self‐pollination. (B) The flowers remained unmanipulated to examine natural pollination. (C) Newly emerged flowers were pollinated by hand with pollen of two or three individuals of the same color and remained uncovered to allow additional pollination by pollinators to gain maximum pollination rate. Flower color is indicated by plot color. Different letters (a, b) indicate statistically significant differences between groups (*P* < 0.05); n.s. = not significant.

Under natural conditions, neither wind‐pollination nor autonomous self‐pollination played a significant role in our study area (for statistics, see Appendix S2: Table S6A). The average relative seed set of bagged individuals (Figure 6A) was <3% in both color morphs, whereas the hand‐pollinated flowers yielded 57 ± 7% relative seed set (Figure 6C). The open‐pollinated flowers showed significantly higher seed set, with 24 ± 7% compared to bagged flowers, but significantly lower seed set compared to open‐pollinated flowers with additional hand pollination (Figure 6B; for statistics, see Appendix S2: Table S6B). Intriguingly, we found very low seed set in red individuals from the open‐pollination treatment of polymorphic populations (6 ± 4%).

**Figure 7:**
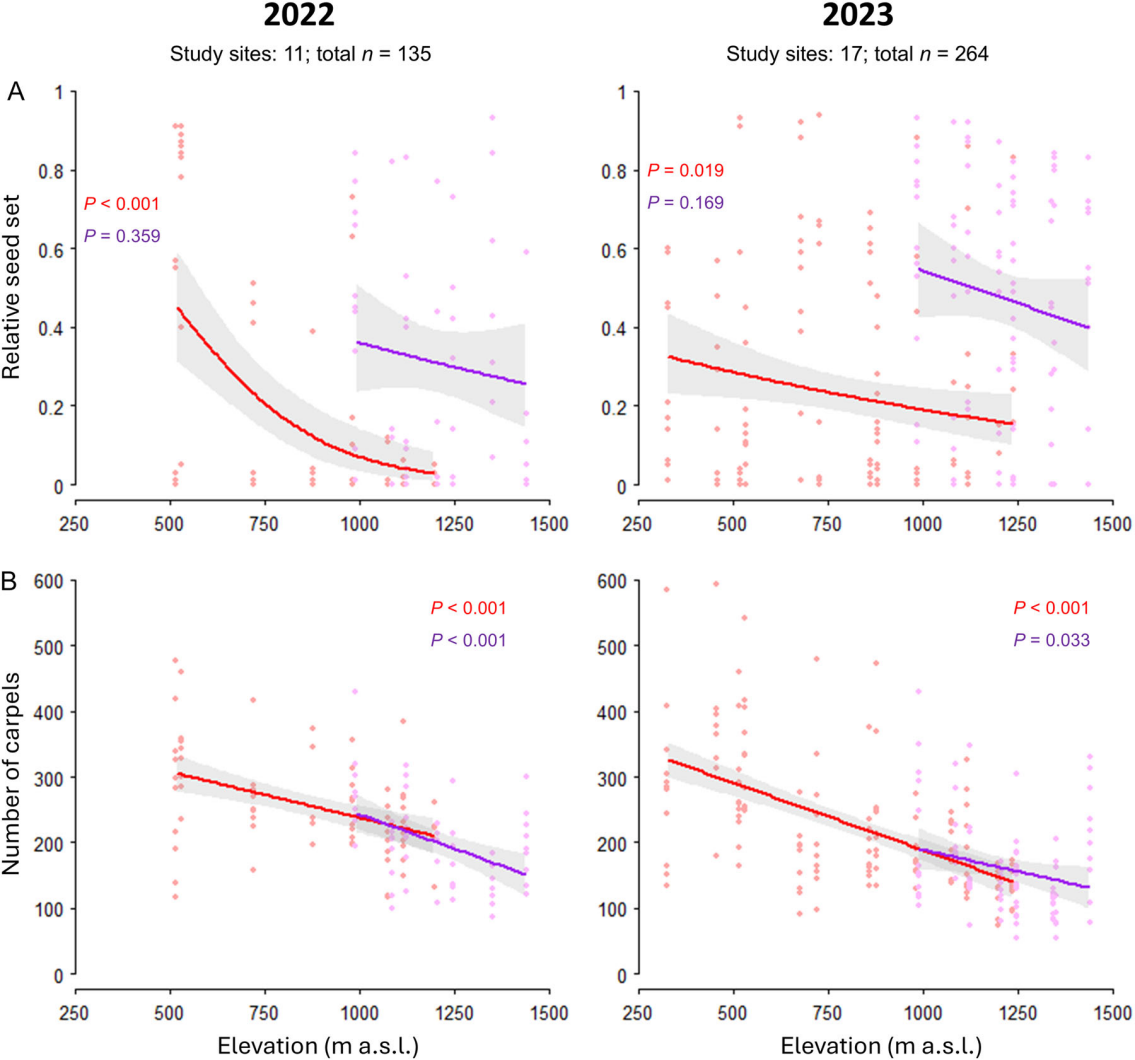
Effect of elevation on (A) relative seed set and (B) number of carpels in *Anemone pavonina* in two consecutive years. Lines show the model adjusted means, gray‐shaded areas show 95% confidence intervals, and dots indicate individual measurements. Flower color is indicated by line or dot color.

We observed a color‐dependent relationship between elevation and relative seed set. Seed set in red individuals decreased with increasing elevation by ~10% every 100 m in both years (2022: *P* < 0.001, df = 67, *R*² = 0.32; 2023: *P* = 0.025, df = 137, *R*² = 0.04; Figure 7A; for statistics, see Appendix S2: Table S7). By contrast, seed set in purple individuals was not affected by elevation (2022: *P* = 0.370, df = 56; *R*² = 0.02; 2023: *P* = 0.148, df = 92, *R*² = 0.02). It should be noted, however, that the elevational distribution range of monomorphic purple populations is much narrower (~250 m) than that of monomorphic red populations (~500 m).

**Figure 8:**
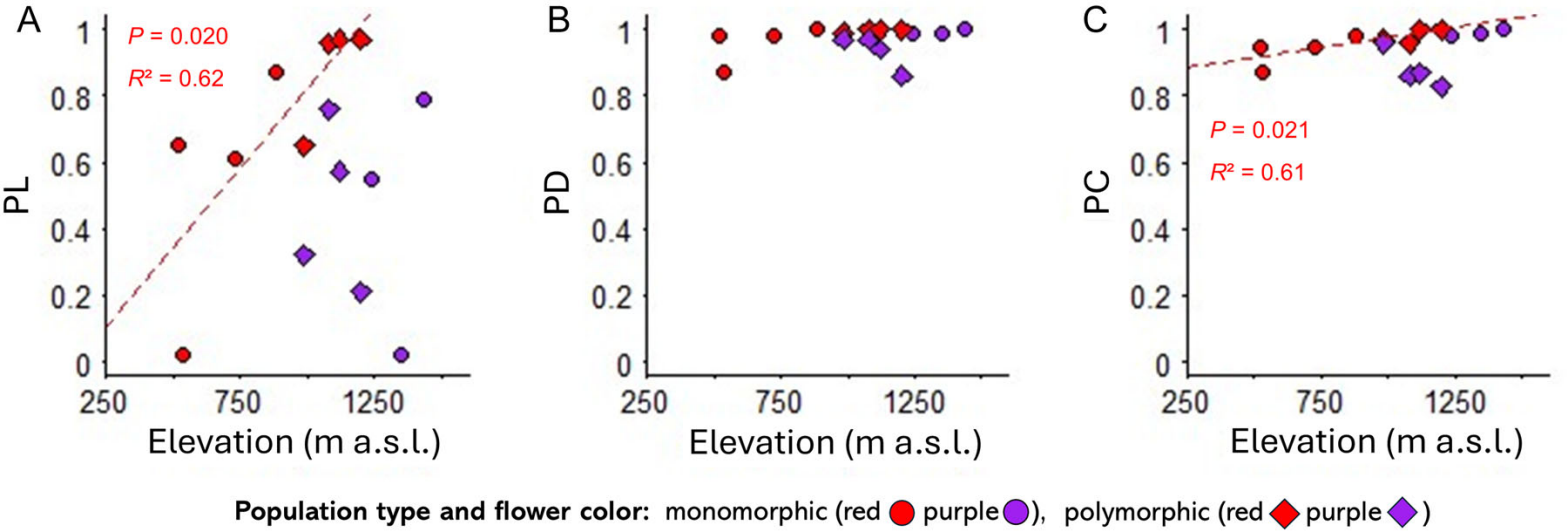
Relationship between elevation and (A) pollen limitation (PL), (B) pollinator dependency (PD), and (C) pollinator contribution standardized to open pollination (PC) in *Anemone pavonina*. Dots show calculated indices per individual study site. Flower color is indicated by dot and line color. Dashed line indicates significant relationship (Appendix S2: Table S12).

The analysis of pollen limitation showed that both morphs are pollen limited (PL = 0.59 ± 0.1). Pollen limitation of the purple morph remained constant across the elevational gradient (PL: monomorphic populations, 0.45 ± 0.23; polymorphic populations, 0.47 ± 0.12; Figure 8A) but increased with elevation in red morphs (PL: monomorphic populations, 0.54 ± 0.18; polymorphic populations, 0.89 ± 0.08; *P* = 0.020, *R*² = 0.62). Across the elevational gradient, both color morphs were highly pollinator dependent (PD: red = 0.98 ± 0.1; purple = 0.96 ± 0.1; Figure 8B), and pollinators contributed almost entirely to seed set (PC: red = 0.96 ± 0.1; purple = 0.93 ± 0.3; Figure 8C).

A comparison of seed set in early‐, mid‐, and late‐season in 2023 revealed that red individuals had the highest seed set during mid‐ and late‐season. Seed set in purple individuals peaked only in mid‐season (Appendix S1: Figure S4; Appendix S2: Tables S9 and S11). The number of carpels declined by 9%–10% per 100 m elevation for both flower color morphs (Figure 7B; Appendix S2: Table S8). We found higher seed set in purple individuals of polymorphic populations in 2023 compared to 2022 (*P* = 0.004), but fewer carpels in both color morphs of polymorphic populations in 2023 than in 2022 (red: *P* = 0.012; purple: *P* < 0.001; Appendix S2: Table S8).

### Pollinator distribution and color preference

**Figure 9:**
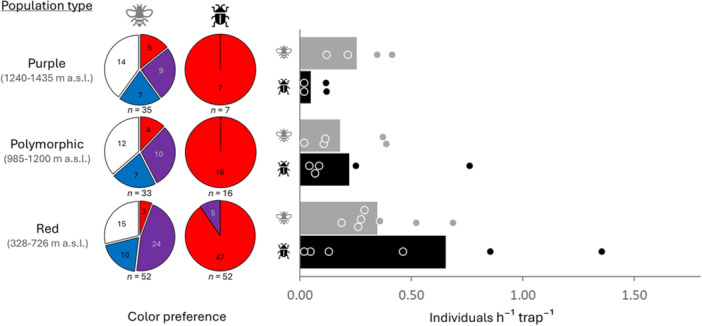
Color preference and distribution of bees (gray) and *Pygopleurus* beetles (black) along the elevational gradient. The individuals were captured in color traps (see Appendix S1: Figure S5) placed in monomorphic red and purple as well as polymorphic populations throughout the *Anemone pavonina* flowering season. The pie charts (left) show the relative proportion of bees and beetles per trap color (number of captured individuals is indicated in each segment, total number of individuals below each chart). The bar graph (right) shows total number of individuals per hour and trap (the bars indicate mean values per population type, dots represent individual study sites). Elevation had a significant effect on pollinator abundance (*P* = 0.004; interactive effect elevation × pollinator group: *P* = 0.05; Appendix S2: Table S12).

In total, 120 bees, 75 beetles (*Pygopleurus* sp.), and 1284 other arthropods were captured in our traps (Appendix S1: Table S2). The number of beetles was highest (0.65 individuals h^−1^ trap^−1^) at low elevations in monomorphic red populations (330–882 m a.s.l.) and decreased by ~8% per 100 m in elevation (*R*² = 0.32, df = 15, *P* = 0.022). In monomorphic purple populations at high elevations (1200–1435 m a.s.l.), only very few beetles were captured (0.05 individuals h^−1^ trap^−1^). By contrast, the number of bees did not differ significantly along the gradient, but it was close to significance (0.18–0.35 individuals h^−1^ trap^−1^; *R*² = 0.25, df = 15, *P* = 0.051). The pan trap catches revealed differences in color preference and distribution of bees and beetles along the investigated elevational gradient (Figure 9; Appendix S2: Tables S9 and S12). Among the 75 beetles, 93% were found in red traps, 7% in purple traps, and none in white or blue traps (Figure 9; cf. Belušič et al., 2025). Beetle color preference did not change with elevation (Appendix S2: Table S12B). By contrast, bees were captured in traps of all four colors, and color preference showed slight but nonsignificant variation along the elevational gradient (Figure 9; Appendix S2: Table S12C). At low elevations, ~50% of the bees were captured in purple traps, compared to 26% at high elevations. White and purple traps were more attractive than red ones at all elevations. Across the elevational gradient, 10% of the bees were captured in red, 20% in blue, 34.2% in white, and 35.8% in purple traps.

## DISCUSSION

The origin and maintenance of flower color polymorphism has fascinated scientists for centuries (reviewed by Narbona et al., 2018; Sapir et al., 2021), but the factors that explain polymorphism can be difficult to uncover (Wright, 1943; Schemske and Bierzychudek, 2007).

Flower color is known to be shaped by multiple environmental factors that may vary temporally and spatially, including climatic conditions (Arista et al., 2013), soil properties (Horovitz, 1976; Landi et al., 2015; Labin et al., 2026), and pollinator availability or behavior (Ortiz et al., 2015; Rodríguez‐Castañeda et al., 2024). Our results demonstrate strong elevation‐related selective pressure that differentially favors specific flower colors. The high pollinator dependence and pollen limitation underscores the role of pollinator‐mediated selection in general (Kay and Anderson, 2025), and in particular as a likely force driving flower color divergence, as different pollinator groups show distinct elevational distributions and color preferences.

We investigated the flowering ecology of the spring‐flowering *A. pavonina*, a species with spatial variation in flower color. We found that *A. pavonina* is protogynous, is self‐compatible, and shows inter‐morph compatibility. However, <5% of seed set resulted from autonomous self‐pollination or wind pollination, while pollinators contributed to >95% of seed set, highlighting the crucial role of insect visitors. Both color morphs experienced pollen limitation across elevations, but pollen limitation increased with elevation in the red and not the purple morph. During the flowering season, bees were predominantly captured in non‐red traps and occurred at similar abundances across the elevational gradient, whereas flower‐visiting *Pygopleurus* beetles showed a preference for red traps and declined in abundance with increasing elevation. We also observed a phenological shift between the morphs: purple flowers started blooming earlier than red flowers, but only in polymorphic populations. This pattern may reflect differential adaptation to the activity of specific pollinators (Ortiz et al., 2015; Rodríguez‐Castañeda et al., 2024) and/or abiotic factors such as temperature (Arista et al., 2013; Dellinger et al., 2026; Grossenbacher et al., 2026), which can affect both morphs differentially (Strauss and Whittall, 2006).

Red and purple *A. pavonina* flowers differed in receptivity over time during the female phase. *Anemone pavonina* has a female phase of 4 days, followed by a male phase of ~8 days. Red flowers exhibited high variation in seed set after hand pollination but had a constant receptivity during the female phase, whereas purple flowers showed a clear decrease in receptivity over time. This might convey a reproductive advantage for red flowers because they have a longer time window to achieve pollination. While we consider it unlikely that the constantly high female receptivity enables red morphs to colonize high elevations where there are few beetle pollinators, high female receptivity may enhance reproductive success at low elevations.

Although the distribution of self‐incompatibility in angiosperms is largely bimodal (Raduski et al., 2012) and might even contribute to flower color polymorphism (Jiménez‐López et al., 2020), there are many species with intermediate levels of self‐compatibility (Lloyd and Schoen, 1992; Good‐Avila and Stephenson, 2002; Raduski et al., 2012). Our results demonstrate that, while *A. pavonina* is partially self‐compatible, it shows almost no auto‐fertility. This may result from dichogamy (the temporal separation of male and female phase) as a mechanism to prevent self‐pollination or may indicate that within‐flower pollen transfer must be facilitated by flower visitors (Lloyd and Schoen, 1992).

Crossbreeding between different *A. pavonina* color morphs is possible, but intermediate flower colors are rare in the field. It remains unclear how color is inherited in *A. pavonina* and whether crossbreeds show purple, red, or intermediate colors. Thus, if hybrids between the red and purple morphs show no reduction in fitness (e.g., infertility, lower seed set, or higher seedling mortality; Hopkins, 2013), this would indicate that the coexistence of the two morphs in polymorphic populations is primarily caused by pollinator‐mediated selection (prezygotic isolation). Inter‐color as well as intra‐color cross‐pollination resulted in equally high seed set, indicating no prezygotic barriers after pollen transfer, as found in the color polymorphic *A. coronaria* (Horovitz, 1995). Postzygotic barriers often manifest in the offspring generations (Widmer et al., 2009; Baack et al., 2015), but it is currently unknown whether *A. pavonina* crossbreeds differ in fitness. However, we did observe seed set in intermediate colors, which are likely to be color crossbreeds, in the field. To clarify this, tests for germination and seedling survival rate for different crosses are currently underway.

The low capacity for autonomous self‐pollination indicates that successful reproduction requires a pollen vector, regardless of whether cross‐ or self‐pollen is deposited on the stigmas. We found that both *A. pavonina* morphs experienced strong pollen limitation across the elevational gradient (Figures 6 and 7). This aligns with previous work indicating that pollen limitation is widespread among entomophilous plants (Ashman et al., 2004; Knight et al., 2005) and a prerequisite for pollinator‐mediated selection (Ashman and Morgan, 2004). Independent of elevation, relative seed set was ~33% lower within the open‐pollination treatment compared to hand pollination. Strikingly, seed set in red but not in purple individuals decreased by ~10% per 100 m increase in elevation, to the level observed in the pollinator‐exclusion treatment. Similarly, pollen limitation increased exclusively in the red morph, indicating that the quantity and/or quality of pollen deposited on red flowers is limited at high elevations. Both morphs exhibited strong pollinator dependency, and pollinators contributed to almost all the seed set (Figure 8) under open pollination across the elevational gradient, highlighting pollinator activity as a key factor in determining morph distribution. We did not observe significant herbivory on leaves or seeds in either morph and therefore consider its effect on plant fitness to be negligible, as reported for *A. coronaria* (Saabna et al., 2025). This may result from the low insecticidal activity of secondary metabolites (Varitimidisa et al., 2006).

Abiotic factors have been shown to affect the reproductive success and distribution of distinct color morphs in different ways, due to color‐related plant traits (Arista et al., 2013; Grossenbacher et al., 2026; Labin et al., 2026). By measuring soil temperature, we started disentangling abiotic factors that are associated with elevation. The number of carpels declined by 9%–10% per 100 m elevational increase (Figure 7B) in both morphs. We hypothesize that the overall reduction in carpel number is driven by changing environmental factors along the elevational gradient, which are typical of mountainous habitats (Körner, 2004). Decreasing temperature (Figure 5) and other factors (e.g., stronger wind or resource limitation) lead to reduced aboveground biomass in plants at high elevations (reviewed by Ma et al., 2010; Halbritter et al., 2018; see also Appendix S1: Figure S7). Given that there is only one ovule per carpel in *A. pavonina*, we conclude that there is a general decline in fitness (maximum seed set) with increasing elevation independent of flower color. Harsher environmental conditions in high‐elevation habitats might also explain the observed decline in seed set in the red morph (e.g., flower color‐dependent maladaptation to altitude or lower stress resistance; Arista et al., 2013) due, for example, to differences in anthocyanin content. However, since hand pollination with additional pollen supply led to high relative seed set in the red morph (equal to that of the purple morph), we argue that the declining seed set is unlikely to be caused by abiotic factors. Instead, a decline in seed set is likely due to the low efficacy and/or absence of suitable pollinators at high elevations, a common phenomenon found in mountainous regions (Warren et al., 1988; Dellinger et al., 2021; McCabe and Cobb, 2021; Izquierdo et al., 2023; Novaes et al., 2024). Although abiotic factors do not seem to have a direct effect on seed set, they may still contribute indirectly to pollen limitation. For instance, ambient temperature likely determines pollinator abundance or activity. Indeed, our results show that beetle abundance and seed set in the red morph strongly decreased with elevation (Figures 7 and 9). *Pygopleurus* beetles should be able to detect all trap colors (Appendix S1: Figure S6C), but they were captured almost exclusively in red traps, presumably because of their strong preference for red colors (Belušič et al., 2025). By contrast, bees were mainly captured in all except red traps, which can be explained by their insensitivity to long wavelength colors (Appendix S1: Figure S6B), and were abundant at all elevations, which would explain the constant pollination level in the purple morph. Intriguingly, there are no white flowers at high elevations, although bees were shown to be attracted to white traps. For this we have several nonexclusive explanations. White *Anemone* flowers may lack anthocyanins, which could decrease stress tolerance and negatively affect seed set or plant growth (Warren and Mackenzie, 2001). Several co‐flowering, white‐flowered species were observed at high elevations. These co‐flowering taxa, such as *Romulea* sp. and *Viola* sp., predominantly offer nectar and may therefore be particularly attractive for nectar‐foraging bees (Muth et al., 2015). Nectar‐foraging bees may thus be attracted to white traps, whereas purple traps may be visited by pollen‐foraging bees, given that pollen is the only reward offered by purple *Anemone* flowers.

Another striking difference between red and purple morphs is the shift in phenology. We found that flowering onset of red individuals in polymorphic populations was delayed by 1.5–2.0 weeks compared to the purple individuals, whereas red and purple individuals in monomorphic populations started flowering at the same time (Figure 5). We therefore assume that temperature is the main trigger for flowering onset in *A. pavonina*, because flowers at low elevations started blooming earlier. Several studies have shown a relationship between temperature and blooming onset (Gilmore and Rogers, 1958; Angus et al., 1981; Heikinheimo and Lappalainen, 1997; McMaster and Wilhelm, 1997; Rojo et al., 2020), suggesting that a certain number of days above a specific temperature is needed to induce species‐specific flowering. Based on this and our pollinator data, we hypothesize that the purple morph has a lower temperature threshold for flowering onset than the red morph, and that the delayed blooming onset of red‐flowered plants in polymorphic populations might even be an adaptation to specific late‐emerging, red‐sensitive pollinators. Indeed, at higher elevations, *Pygopleurus* beetles occur rather late in the season (Appendix S1: Figure S8; Bollino et al., 2019), certainly later than bees, presumably because their emergence follows rising temperatures uphill. Overall, our findings imply that the purple morph is better adapted to low temperatures and early‐occurring pollinators (such as bees), whereas red flowers rely on glaphyrid beetles that are abundant later in the season (Keasar et al., 2008). Taken together, this suggests that pollinator‐mediated selection, driven by differential color preferences and spatiotemporal patterns of bee and beetle pollinator abundance, shapes the distribution of flower color morphs.

However, some questions remain: What explains the comparatively low proportion of intermediate color morphs, and why do purple individuals not invade monomorphic red populations at lower elevations where bees occur? Importantly, detailed information about the behavior of flower‐visiting bees and beetles is currently lacking (e.g., color constancy, pollination effectiveness, and visitation rates). We thus encourage further experiments to address the roles of bee and beetle pollinators (Johnson et al., 2020; von Witt et al., 2020), particularly their pollination effectiveness, behavior during flower visitation, and pollen transmission rates (Ne'eman et al., 2010; Koski et al., 2018). In addition, the influence of abiotic factors, such as soil properties (Schemske and Bierzychudek, 2007) and temperature (Arista et al., 2013), requires further study—especially in regard to seedling survival.

## CONCLUSIONS

Several traits appear to be crucial for the maintenance of flower color polymorphism in *A. pavonina*. Red and purple flowers differed in pollen limitation and phenology along the elevational gradient. The red morph experienced stronger pollen limitation, lower seed set, and delayed onset of flowering in polymorphic populations. The elevation‐dependent decline in seed set and the phenological shift of the red morph correspond to the distribution and activity period of its primary pollinators (glaphyrid beetles), whereas purple flowers maintained relatively constant levels of pollen limitation and seed set, likely because of the consistent presence of bees across elevations.

Together, these findings illustrate how spatiotemporal dynamics of abiotic and biotic factors—and their interactions—shape floral trait variation through flower color‐associated plant characteristics. Our study further highlights potential factors underlying the emergence of flower color variation and provides insight into the evolution and maintenance of flower color polymorphism.

## AUTHOR CONTRIBUTIONS

J.H., J.S., and G.V. designed the experiments. J.H. performed the experiments, with the help of J.S. and C.J.v.d.K. U.J. collected data and took care of the plants at the Botanical Garden. J.H. analyzed the data. J.H. wrote the first draft of the manuscript. J.H., J.S., C.J.v.d.K., and G.V. edited the manuscript. All authors approved the submitted version.

## CONFLICT OF INTEREST STATEMENT

The authors declare no conflict of interest.

## Supporting information


**Appendix S1.** Additional data on study sites, floral ecology, trap details and catches, and pollinator color space.

**Appendix S2.** Tables containing all statistical tests or models used and their results.

## Data Availability

All data for this study can be found within the manuscript, the supporting materials, and online at Figshare: https://doi.org/10.6084/m9.figshare.31301656.
