# Supplementary material for: Flower color polymorphism in the peacock anemone (Anemone pavonina) reflects spatiotemporal variation in pollinator abundance
Source: Am J Bot. 2026 Apr 8;113(4):e70189. doi: 10.1002/ajb2.70189 (PMC13103634; doi:10.1002/ajb2.70189)
Supplement: Supplementary file 1 — Appendix S1. Additional data on study sites, floral ecology, trap details and catches, and pollinator color space. [file AJB2-113-e70189-s001.pdf]

Study site details

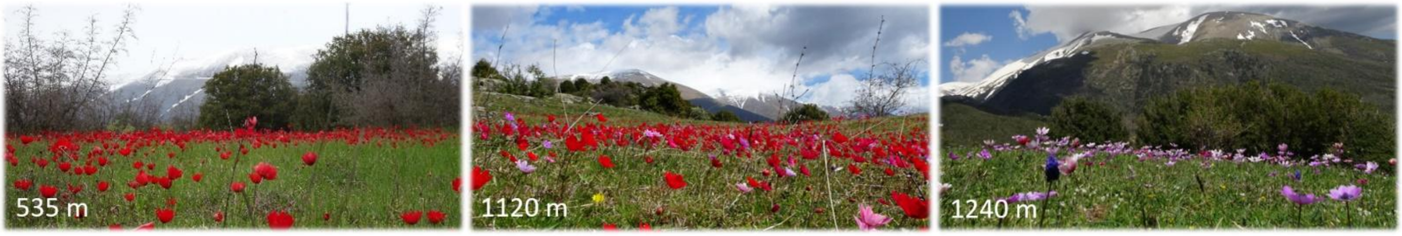

Table S1: Detailed description of study site parameters used for our experiments. Proportion refers to the percentage of purple, intermediate and red flowers within polymorphic populations. Study sites highlighted in grey are used in the extended study design in 2023. On these sites transect walks or temperature measurements did not take place.

Site	Type	Flower colour	Elevation [m a.s.l.]	Population size (individuals)	Distance to next site	Morph proportion purple	inter-mediate	red	Mean temperature °C (November'22 – April'23)
**2022_R5**	monomorphic	red	520	> 1000	2.5 Km			1	8.3
**2022_R6**	monomorphic	red	535	> 1000	2.5 Km			1	9.6
**2022_R1**	monomorphic	red	726	> 1000	2.1 Km			1	8.1
**2022_R11**	monomorphic	red	882	> 500	1.0 Km			1	8.8
**2022_M7**	polymorphic	purple, red, intermediate	985	> 1000	2.4 Km	0.24	0.18	0.59	5.5
**2022_M2**	polymorphic	purple, red, intermediate	1080	> 1000	0.8 Km	0.64	0.24	0.12	7.8
**2022_M9**	polymorphic	purple, red, intermediate	1120	> 1000	0.8 Km	0.47	0.28	0.25	7.0
**2022_M10**	polymorphic	purple, red, intermediate	1200	> 500	0.4 km	0.65	0.24	0.11	7.7
**2022_P4**	monomorphic	purple	1240	> 1000	0.4 Km	1			5.7
**2022_P8**	monomorphic	purple	1348	> 1000	2.2 Km	1			6.1
**2022_P3**	monomorphic	purple	1435	> 1000	0.8 km	1			6.4
**2023_R17**	monomorphic	red	328	> 1000	12.4 km				
**2023_R16**	monomorphic	red	460	> 500	7.8 km				
**2023_R13**	monomorphic	red	680	> 1000	5.2 Km				
**2023_R14**	monomorphic	red	863	> 500	1.8 Km				
**2023_M15**	polymorphic	red, purple, intermediate	1240	> 500	0.6 Km				
**2023_P12**	monomorphic	purple	1339	> 1000	0.4 Km				

Pollinator monitoring

Table S2: List of all individuals caught in the pan traps (purple, red, blue, white) within three consecutive years. Some beetles were identified to family or genus level to evaluate their importance as putative pollinators.

Site	Population type	Trap color	Elevation	Sampling hours	*Pygopleurus sp.*	*Tropinota sp.*	*Eulasia sp.*	Cleridae	Other beetles	Apiformes	Apocrita	Symphyta	Formicidae	Other Hymenoptera	Nematocera	Brachycera	Heteroptera	Orthoptera	Sternorrhyncha	Auchenorrhyncha	Lepidoptera	Arachnida	Psocoptera	Sum
2024_Hel	red	purple	320	5						1														1
2024_Hel	red	red	320	5	6		1			1														8
2024_Hel	red	blue	320	5		3																		3
2024_Hel	red	white	320	5		2			1	1														4
2024_Pp_R5	red	purple	500	6					1															1
2024_Pp_R5	red	red	500	6	5	2				1														8
2024_Pp_R5	red	blue	500	6		4																		4
2024_Pp_R5	red	white	500	6					1															1
2022_R5	red	purple	520	54	2	4			50	6			1	1		2		1		1				68
2022_R5	red	red	520	54	4			1	1				1		1	1			2					11
2022_R5	red	blue	520	54		4			64	4				1		1			2					76
2022_R5	red	white	520	54					13	3				1	4	3			1					25
2022_R6	red	purple	535	38		1			43	4			2	1		3								54
2022_R6	red	red	535	38	1		1		4				2	1		2			2			1		14
2022_R6	red	blue	535	38		5	1		43	1			1		1	3	1		1					57
2022_R6	red	white	535	38		1			29	8					1	6			1					46
2024_Ela	red	purple	550	6		3				3														6
2024_Ela	red	red	550	6	11		5																	16
2024_Ela	red	blue	550	6		1	1		2															4
2024_Ela	red	white	550	6		1																		1
2022_R1	red	purple	726	52	3	9			32	7	3		3		2	3			2	1				65
2022_R1	red	red	726	52	20	3			8	1			3	1	2	1	1		1		1			42
2022_R1	red	blue	726	52		5		2	25	3	1		3	2	7	4	3		2	1				58
2022_R1	red	white	726	52					8	3			3	2	4	2	1		1			2		26
2022_R11	red	purple	882	20					6	3					6	1								16
2022_R11	red	red	882	20					2		1				3	1								7
2022_R11	red	blue	882	20		1			8	2				1	4	3								19
2022_R11	red	white	882	20					1		1				3	4								9
2022_M7	polymorphic	purple	985	48					11	5	1				4	3	1		2	2	1			30
2022_M7	polymorphic	red	985	48	1			1	3	4	2			3	5	5			4	1				29
2022_M7	polymorphic	blue	985	48		5			19	4	3		2	1	4	1			5	1		1	1	47
2022_M7	polymorphic	white	985	48					9	4	2		3	1	3	5			2			2	2	33
2022_M2	polymorphic	purple	1080	46					4	2	1				1				1	2				11
2022_M2	polymorphic	red	1080	46	3							2	8		1	1			2		1			18
2022_M2	polymorphic	blue	1080	46									2		2	1			1	1				7
2022_M2	polymorphic	white	1080	46					3	2			1			1		1	1		1			10
2022_M9	polymorphic	purple	1120	43		1			1	1	1	1	1		1				12					19
2022_M9	polymorphic	red	1120	43	2				1				1			3			12					19
2022_M9	polymorphic	blue	1120	43					6	2			1		1	1			10					21
2022_M9	polymorphic	white	1120	43					7	1			1		5	2			7				2	25
2022_M10	polymorphic	purple	1200	22		1			6	2	1				8	7			3			1	1	30
2022_M10	polymorphic	red	1200	22	5	1			8					2	6	3	2		2		1			30
2022_M10	polymorphic	blue	1200	22		1			8	1	3		2		5	9								29
2022_M10	polymorphic	white	1200	22					7	5	1				5	12			3					33
2023_M15	polymorphic	purple	1240	51												1								1
2023_M15	polymorphic	red	1240	51	5											1					1			7
2023_M15	polymorphic	blue	1240	51										1		1								2
2023_M15	polymorphic	white	1240	51																				0
2022_P4	purple	purple	1240	7					7	5	1	1	1		5	6			2					1
2022_P4	purple	red	1240	7	5				7	4			2		1	9	2		5	1				7
2022_P4	purple	blue	1240	7					6	4	1		2		4	4			3			3		2
2022_P4	purple	white	1240	7					6	7	2				3	12	1		2		1	1		0
2023_P12	purple	purple	1338	20					3	1				1										5
2023_P12	purple	red	1338	20	2				5															7
2023_P12	purple	blue	1338	20		1			1	1						1								4
2023_P12	purple	white	1338	20		1			3	2							1							7
2022_P8	purple	purple	1348	15					1	2	1					10			3					17
2022_P8	purple	red	1348	15					3				3		3	5			5					19
2022_P8	purple	blue	1348	15								1			1	6			2					10
2022_P8	purple	white	1348	15					1	3					2	9			1			1		17
2022_P3	purple	purple	1435	61		1			10	1			2	1	10	12			12				2	51
2022_P3	purple	red	1435	61			1		19	1	1				12	19	1		10			1		65
2022_P3	purple	blue	1435	61					8	2	1	1		1	13	17	2		6	1	1		1	54
2022_P3	purple	white	1435	61					6	2			1	2	8	16	1		10					46
			Sum	1977	75	61	10	4	521	120	28	6	52	24	151	223	17	2	143	12	8	13	9	

Basic floral ecology

Pistil and anther development

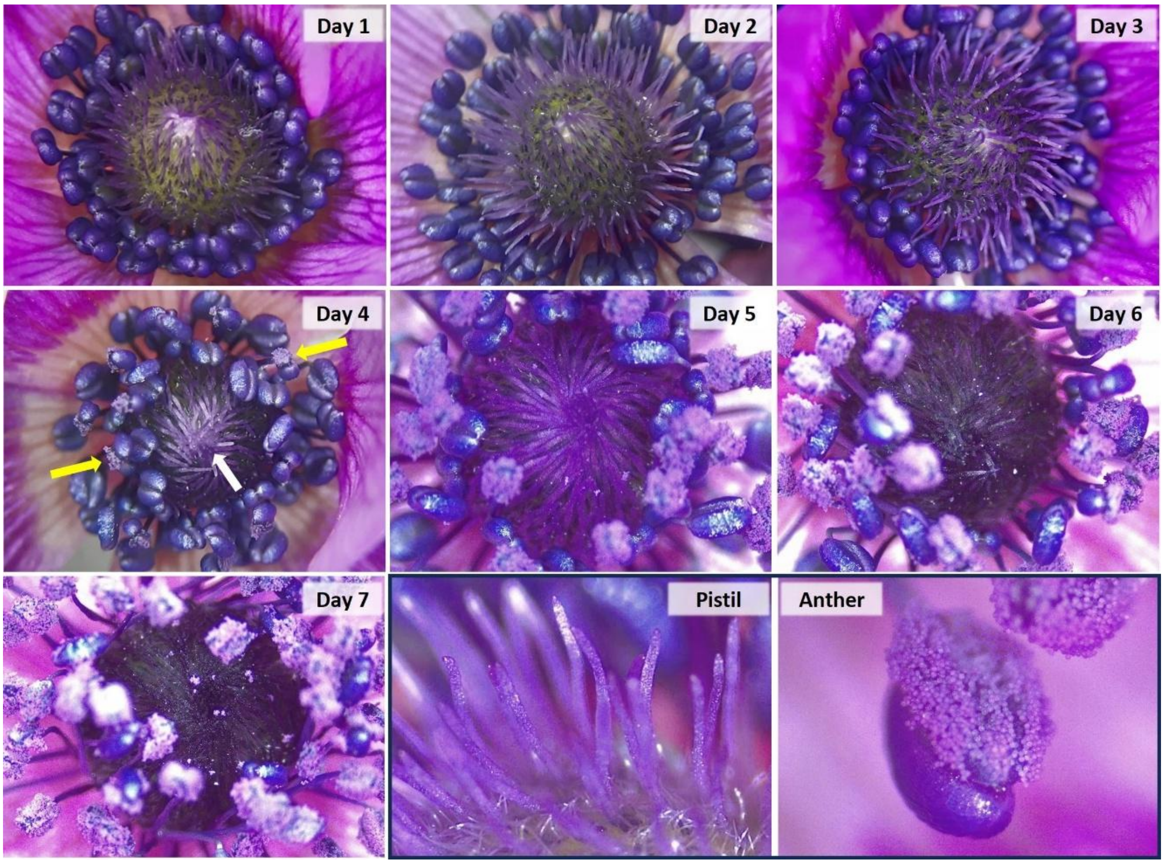

Figure S1: Pistil and anther development, and pollen release in a purple *A. pavonina* flower **(**only the first seven days are shown. The male phase typically ends after ca. 8–12 days, as the plant begins to shed its petals and anthers. Eventually, only the developing infructescence remains). Within the first three days the pistils bend outwards in all directions. At day four, the pistils bend to the centre (white arrow). The pistil colour changes from pale to dark purple over time (cf. day 5 and 7). Anther dehiscence starts on day four (yellow arrow) and continues over the next few days. The close-ups show pistils at day one and pollen of a six days old flower.

Anthesis and number of carpels

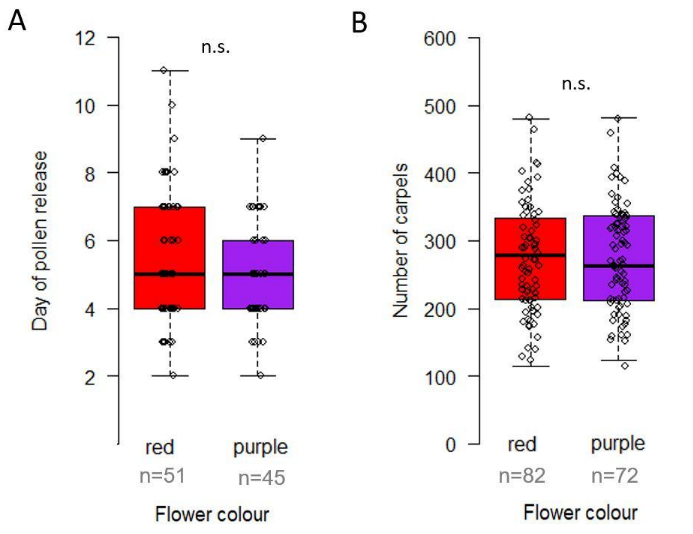

Figure S2: Comparison of day of pollen release (dehiscence) (**A**) and number of carpels (**B**) between flowers of red and purple individuals (for statistics see Appendix S2, Tables S2 and S3).

Temporal characteristics of fecundity

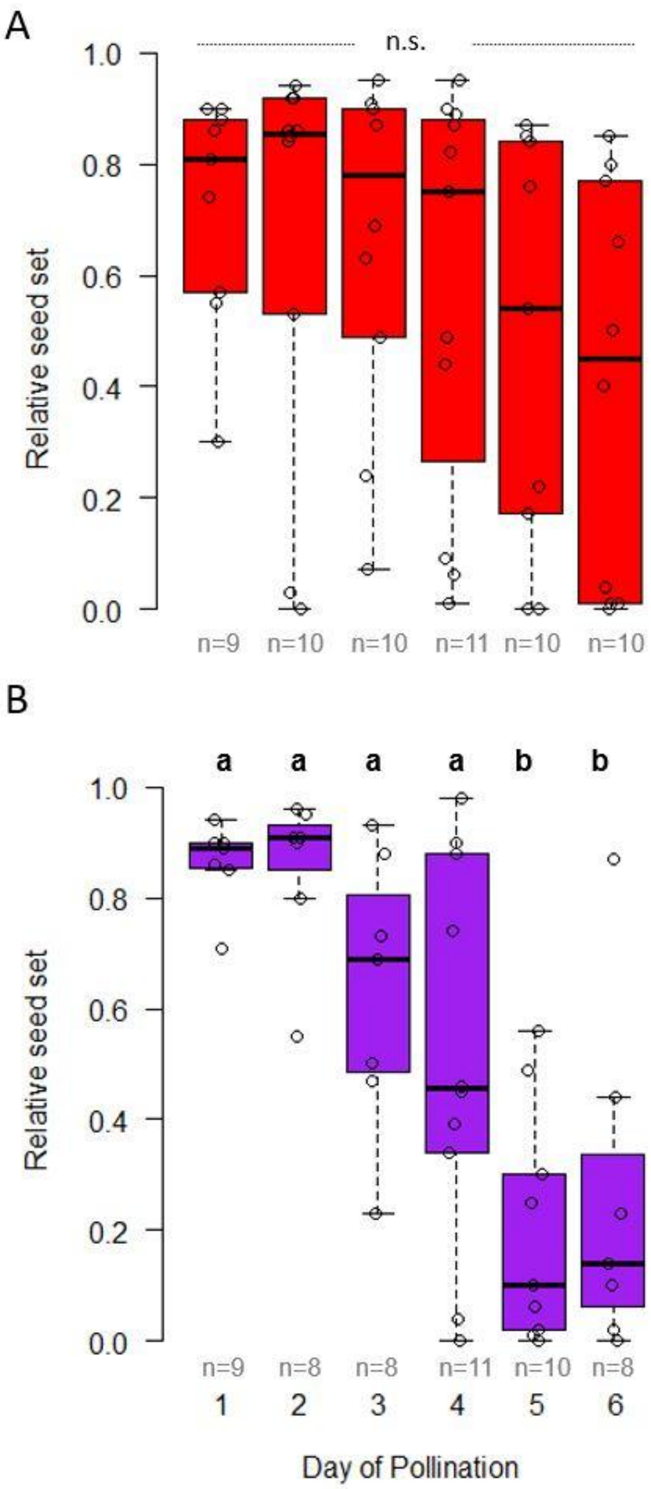

Figure S3: Relative seed set dependent on day of hand pollination – with pollen of two to three individuals of the same colour – in red (**A**) and purple (**B**) individuals (for statistics see Appendix S2, Tables S2 and S3).

Temporal variation in seed set

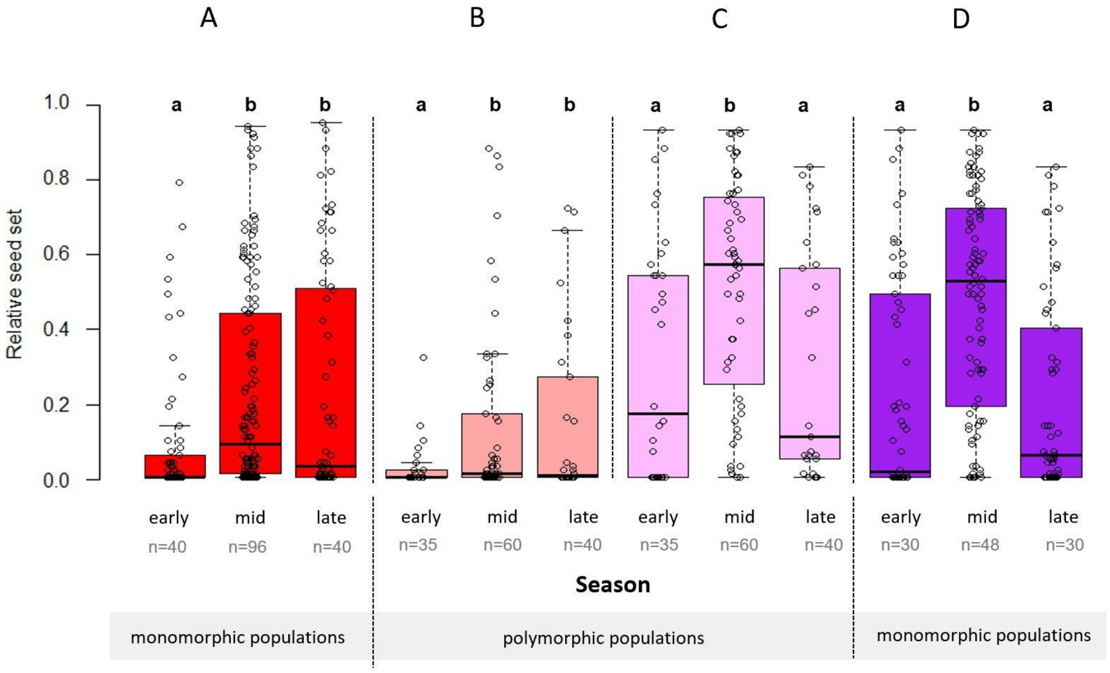

Figure S4: Relative seed set in the open pollination treatment of red and purple individuals of monomorphic and polymorphic populations for different times during the season. Start seasonal phases: **early**: April 7th; **mid**: April 21st; **late**: May 4th.

Pollinator monitoring

Pan traps

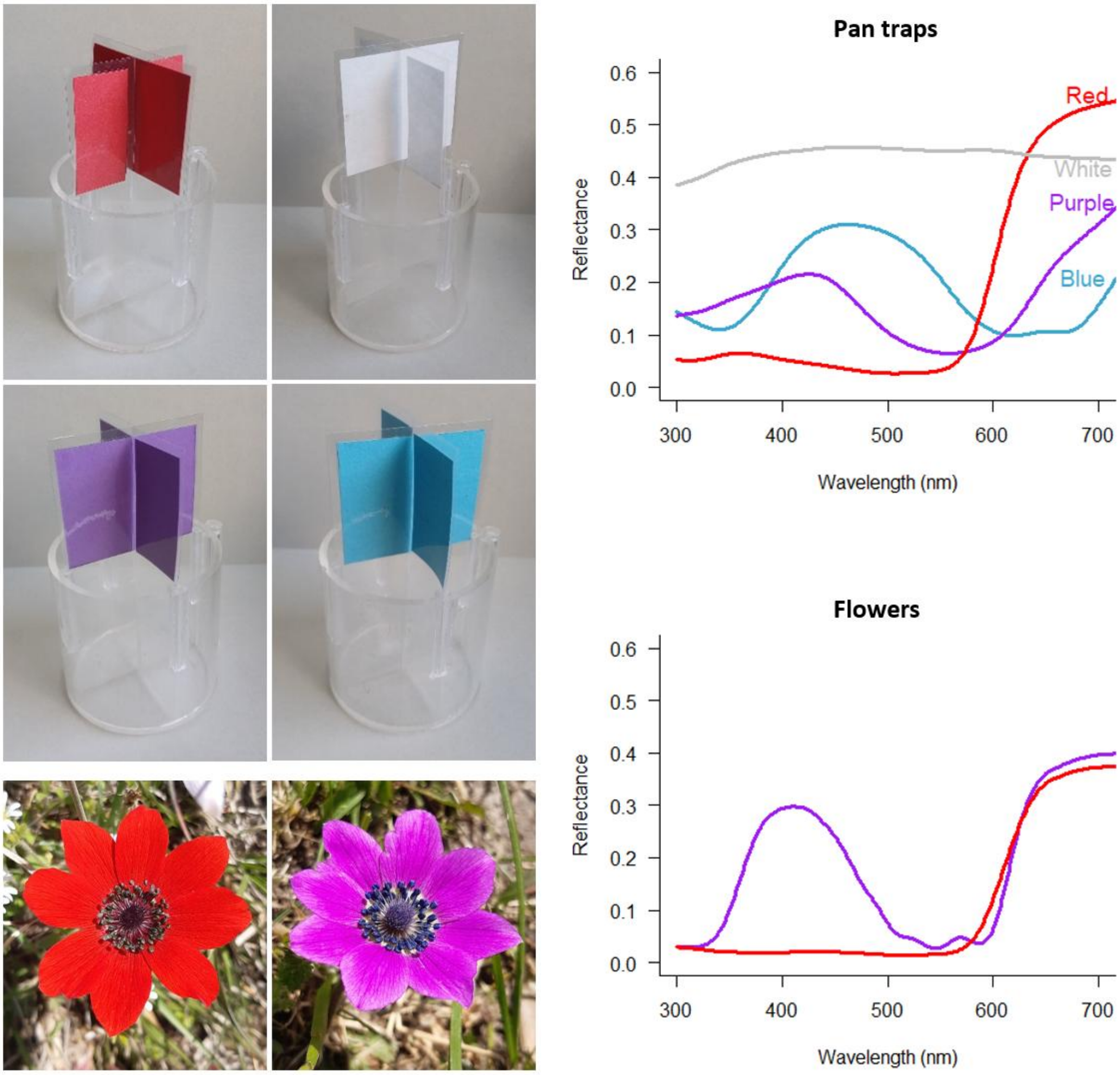

Figure S5: Left: Pictures of the four differently coloured pan traps (dimension: traps: ø 7 cm, h = 7 cm; cross-vanes: h =12 cm, w = 6 cm; coloured part of the cross-vanes: h = 5 cm, w = 5.5 cm) and the two main colour morphs of *A. pavonina.* As colour stimuli tinted drawing paper (Buntpapierfabrik Ludwig Bähr, Kassel, Germany) was used: purple (#217 46 62), red (#217 46 22), blue (#217 46 31). As white stimulus we used filter paper circles (No. 595, ø 125mm, Schleicher & Schuell, Dassel, Germany). The tinted paper was laminated with clear 80 micron laminating foil (#510361, 3T Supplies AG Peach Division, Schindellegi, Switzerland). **Right:** Reflectance spectra of traps and *A. pavonina* flowers. The reflectance spectra were measured with a bifurcated probe under an angle of ~30 degrees to avoid surface reflections (see Belušič et al., 2025).

<u>Pollinator colour space</u>

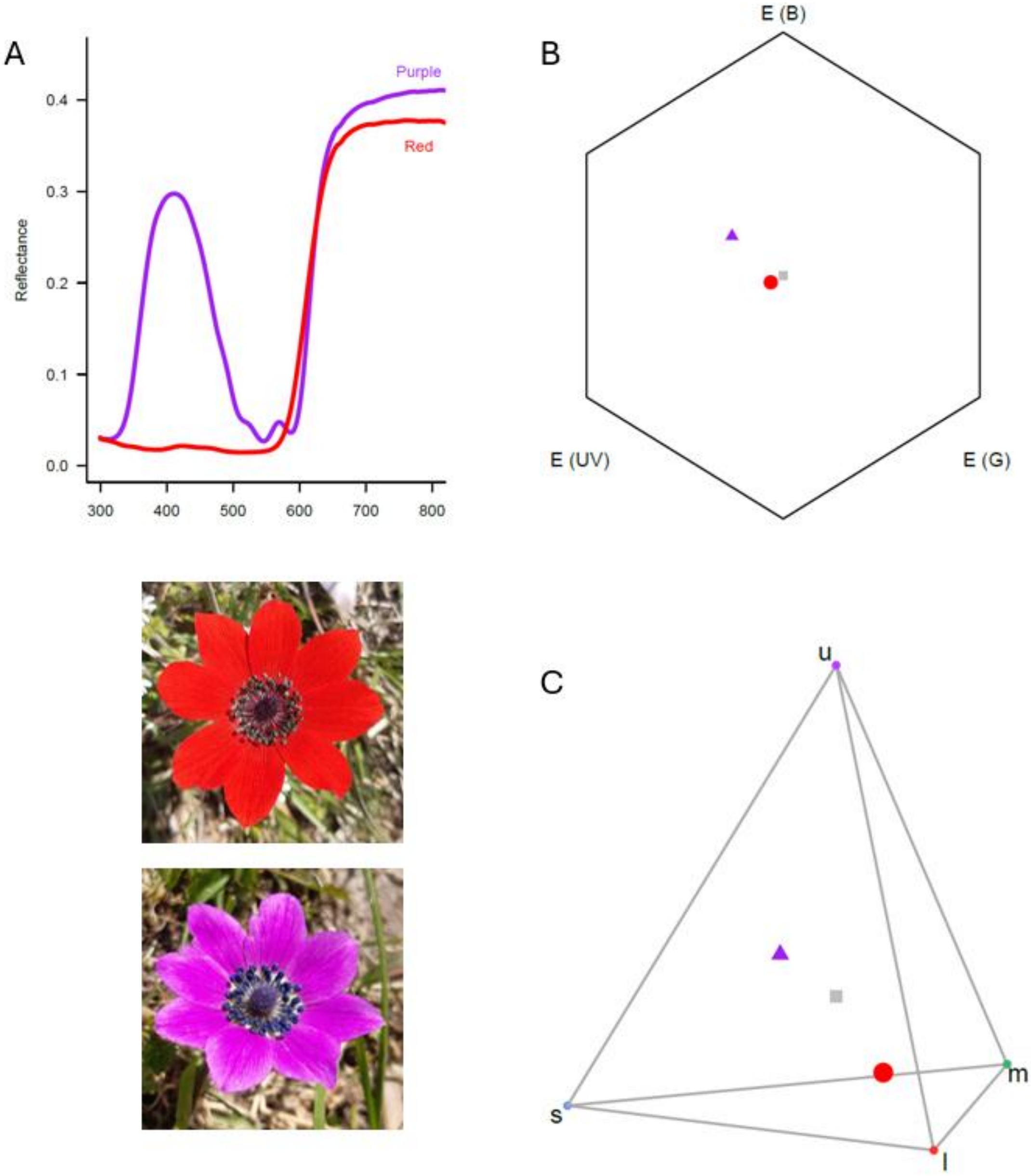

Figure S6: Reflectance spectra of red and purple *A. pavonina* flowers (A). The plots on the right show the positioning of the red (red circle) and purple (purple triangle) flowers in honeybee hexagon colour space (B) and *Pygopleurus* beetle tetrahedron space (C), respectively. Colour contrast values against a green background in D65 illumination are 0.28 for purple and 0.06 for red flowers in the hexagon. In the tetrahedron, colour contrast against a green background is 0.18 for purple and 0.42 for red flowers. For calculation see León-Osper and Narbona (2022), photo receptor sensitivities of *Pygopleurus* were taken from Belušič et al. (2025).

Elevational effects

Flower diameter-elevation relationship

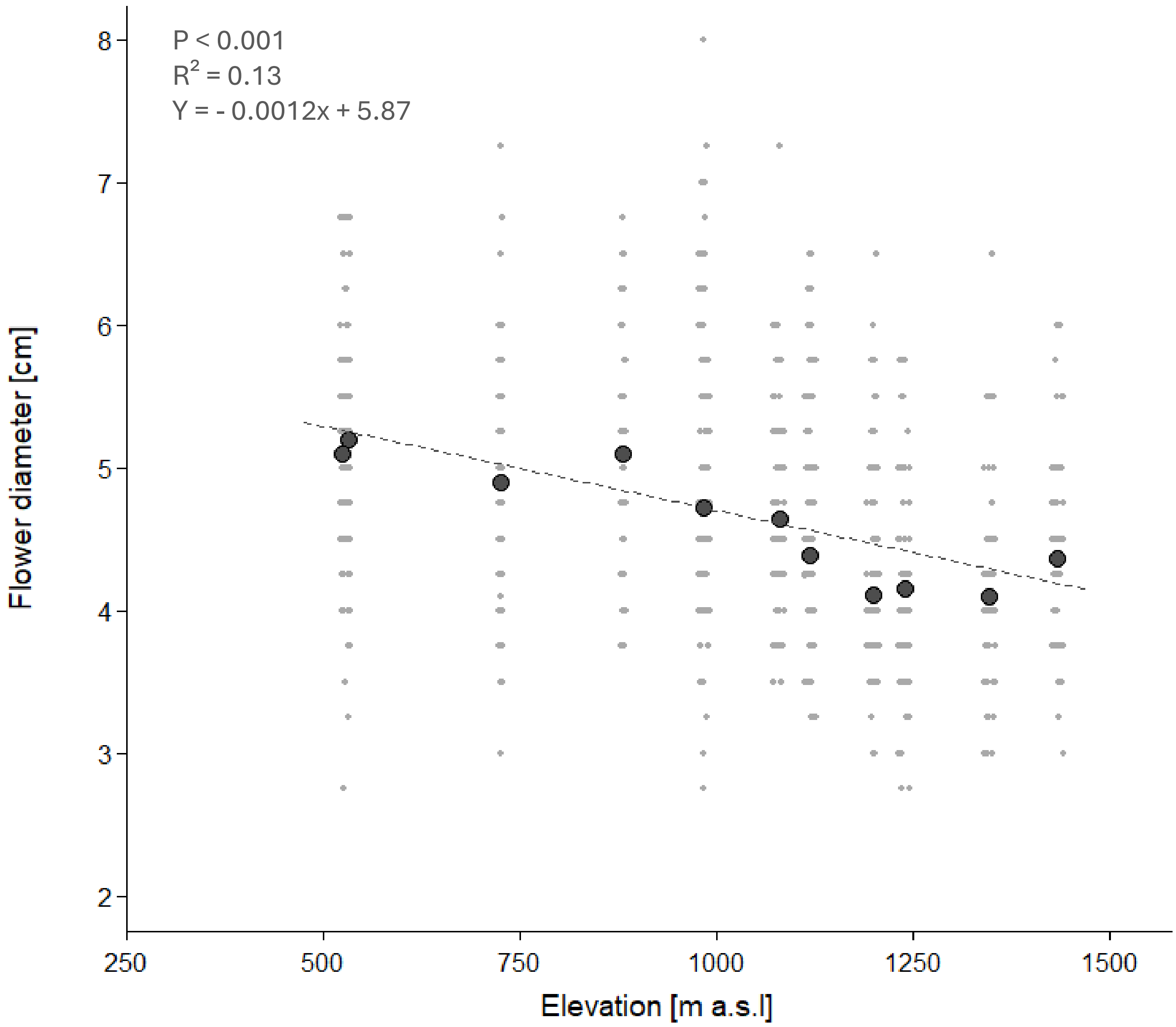

Figure S7: To examine flower diameter, we measured more than 800 *A. pavonina* flowers (red and purple; age 1-2 days) within eleven populations in Greece along an elevational gradient from 525m to 1435m a.s.l. We found that flower size decreased with elevation (1.2mm per 100m) independent of flower colour. However, this relationship is weak ($R^2 \leq 0.13$). Large dots indicate the mean flower diameter per site; small dots indicate the individual measurements of each flower.

Pollinator phenology across the elevational gradient

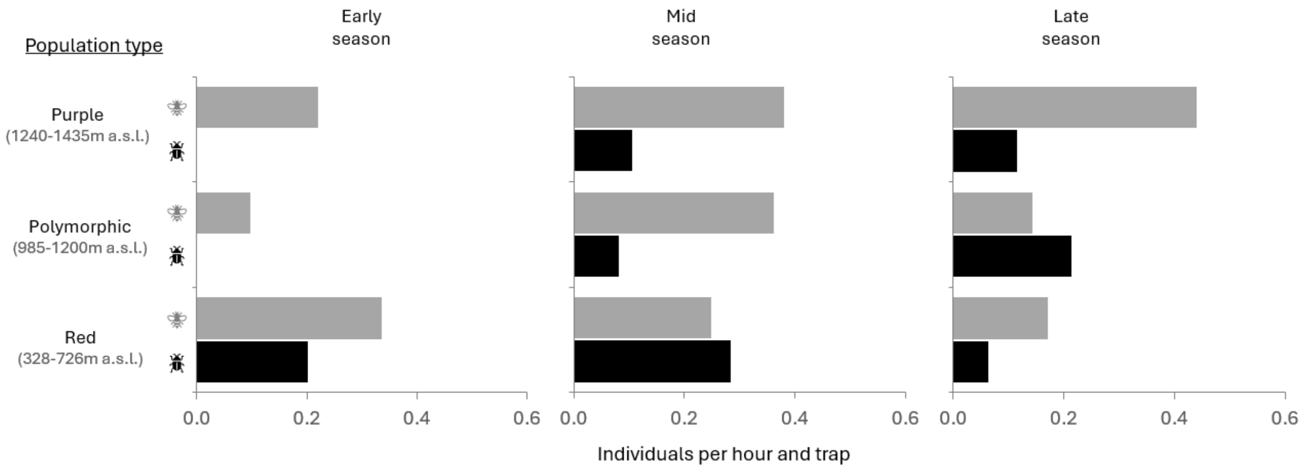

Figure S8: Pollinator abundance across the elevational gradient for each population type in the early, mid-, and late season, based on pan-trap data. Start seasonal phases: **early**: April 7th; **mid**: April 21st; **late**: May 4th. Bars indicate the number of individuals per hour per trap (black: *Pygopleurus*; grey: bees).

References

Belušič G, de Hoop SB, Bencúrová E, Lazar D, Spaethe J, van der Kooi CJ. 2025. Remarkable red colour vision in two Mediterranean beetle pollinators. *Journal of Experimental Biology* 228(12): jeb250181.

León-Osper M, Narbona E. 2022. Unravelling the mystery of red flowers in the Mediterranean Basin: How to be conspicuous in a place dominated by hymenopteran pollinators. *Functional Ecology* 36: 2774-2790.
