# Supplementary material for: Flower color polymorphism in the peacock anemone (Anemone pavonina) reflects spatiotemporal variation in pollinator abundance
Source: Am J Bot. 2026 Apr 8;113(4):e70189. doi: 10.1002/ajb2.70189 (PMC13103634; doi:10.1002/ajb2.70189)
Supplement: Supplementary file 2 — Appendix S2. Tables containing all statistical tests or models used and their results. [file AJB2-113-e70189-s002.pdf]

Table S1: Overview of statistical tests and methods used for analysis.

Description of factors and possible values (**flower colour:** red, purple; **population type:** red, polymorphic, purple; **Season:** early, mid, late; **pollination treatment:** hand, open, covered; **breeding system:** cross-pollination, geitonogamous pollination, autonomous self-pollination; **cross-pollination type** [ovule-pollen]**:** red-red, red-purple, purple-red, purple-purple; **pollinator groups**: bees, beetles). The R-code follows the structure: dependent variable ~ independent variable1*Independent variable2*…+ random factor. * Indicates that the interaction between factors is included. If no more details are provided, we used the default values for each command/ test/ package.

Experiment	Comparison of…	Statistical test	Distribution/ link function	R command [package]	R-code/ variables
Number of carpels	**Number of carpels** between red and purple flowers	Wilcoxon test		wilcox.test [stats]	Number_of_carpels~Flower_colour
Day of pollen release	**Day of pollen release** between red and purple flowers	Wilcoxon test		wilcox.test [stats]	Day_of_pollen_release~ Flower _colour
Day of pollination	**Relative seed set** depending on **day of pollination** (colour morph intern)	Kruskal-Wallis test + Dunn post-hoc with Bonferroni correction		kruskal.test [stats] + dunn_test [rstatix]	Relative_seed_set~Pollination_day
Breeding system	**Relative seed** set depending on **pollination treatment** (for each colour morph)	Kruskal-Wallis test + Dunn post-hoc with Bonferroni correction		kruskal.test [stats] + dunn_test [rstatix]	Relative_seed_set~Pollination_treatment
Inter-colour compatibility (cross-pollination between both colour morphs)	**Relative seed** set between **intra and inter morph crosses**	Kruskal-Wallis test + Dunn post-hoc with Bonferroni correction		kruskal.test [stats] + dunn_test [rstatix]	Relative_seed_set~Cross_pollination_type
Pollinator exclusion experiment	Differences in **relative seed set** depending on **pollination treatment, flower colour** and **population type**	Generalized Linear Mixed Model	**Quasibinomial** (relative seed set is proportional data; values between 0 and 1)	glmmPQL [MASS] + emmeans [emmeans] + Anova [car] for summary	Relative_seed_set~Treatment*Flower_color *Population_type,random=~1|Site
Seasonal effects	Differences in **relative seed set** depending on **population type, flower colour** and **seasonality**	Generalized Linear Mixed Model	**Quasibinomial** (relative seed set is proportional data; values between 0 and 1)	glmmPQL [MASS] + emmeans [emmeans] + Anova [car] for summary	Relative_seed_set~Season* Flower_color *Population_type,random=~1|Site
Relative seed set along the elevational gradient	Differences in **relative seed set** depending **on flower colour, population type** and **year**	Generalized Linear Mixed Model	**Quasibinomial** (relative seed set is proportional data; values between 0 and 1)	glmmPQL [MASS] + emmeans [emmeans] + Anova [car] for summary	Relative_seed_set~Flower_color *Population_type*Year,random=~1|Site
Number of carpels along the elevational gradient	Differences in **number of carpels** depending on **flower colour, population type** and **year**	Generalized Linear Mixed Model	**Gaussian**	glmmPQL [MASS] + emmeans [emmeans] + Anova [car] for summary	Number of carpels~ Flower_color *Population_type*Year,random=~1|Site

Pollinator dependency along the elevational gradient	Relationship between **pollinator dependency** and **elevation** (for each colour morph)	Generalized linear model, linear regression	**Gaussian**	glm [stats]	PD~Elevation
Pollen limitation along the elevational gradient	Relationship between p**ollen limitation** and **elevation** (for each colour morph)	Generalized linear model, linear regression	**Gaussian**	glm [stats]	PL~Elevation
Pollinator contribution along the elevational gradient	Relationship between **pollinator contibution** and **elevation** (for each colour morph)	Generalized linear model, linear regression	**Gaussian**	glm [stats]	PC~Elevation
Flowering peak	Differences in **flowering peak** with respect to **population type** and **flower colour**	Linear mixed model	**Gaussian**	lmer [lme4] + emmeans [emmeans]	Flowering_peak~Population_ type*Flower_colour+(1|Site)
Flowering peak	Relationship between **flowering peak** and **elevation** (colour morph intern)	Generalized Linear model, linear regression	**Gaussian**	glm [stats]	Flowering_peak~Elevation
Temperature gradient	Relationship between **temperature** and **elevation**	Generalized Linear model, linear regression	**Gaussian**	glm [stats]	Temperature~Elevation
Pollinators	Effect of **elevation** and pollinator group on **number** of caught pollinators per hour and trap	Linear mixed model	**Gaussian**	lmer [lme4] + emmeans [emmeans]	Individuals_per_hour_trap~ Population_ type*Pollinator group+(1|Site)
Pollinators	Relationship between **number of pollinators caught** per hour and trap and **elevation** (for both pollinator groups separately)	Generalized Linear model, linear regression	**Gaussian**	glm [stats]	Individuals_per_hour_trap~Elevation
Pollinators	Relationship between **number of pollinators caught** and **trap colour** and **population type** (for both pollinator groups separately)	Linear mixed model	**Gaussian**	lmer [lme4] + emmeans [emmeans]	Individuals_caught~ trap_colour*Population_ type+(1|Site)

Table S2: Statistical results (for Appendix S1, Figure S3B) on the effect of flower age of purple flowers (at the moment of pollination) and relative seed set in purple flowers (Kruskal-Wallis test). Significant results are indicated in bold.

Flower colour: purple (Figure S4B)

Trait	Factor	**p-value**	**Chi²**	**df**
**Relative seed set**	**Day of pollination**	**<0.001**	24.23	5

Dunn's post-hoc (Bonferroni)	**p-value**
Day1 – Day2	0.704
Day1 – Day3	0.250
Day1 – Day4	0.069
Day1 – Day5	**0.009**
Day1 – Day6	**0.036**
Day2 – Day3	0.126
Day2 – Day4	0.385
Day2 – Day5	**0.002**
Day2 – Day6	**0.010**
Day3 – Day4	0.568
Day3 – Day5	0.418
Day3 – Day6	0.898
Day4 – Day5	0.072
Day4 – Day6	0.142
Day5 – Day6	0.203

Table S3: Statistical results (for Appendix S1, Figure S3A) on the effect of flower age of red flowers (at the moment of pollination) and relative seed set in red flowers (Kruskal-Wallis test). Significant results are indicated in bold.

Flower colour: red (Figure S4A)

Trait	Factor	**p-value**	**Chi²**	**df**
**Relative seed set**	**Day of pollination**	0.124	8.63	5

Dunn's post-hoc (Bonferroni)	**p-value**
Day1 – Day2	0.966
Day1 – Day3	0.987
Day1 – Day4	0.499
Day1 – Day5	0.117
Day1 – Day6	0.561
Day2 – Day3	0.943
Day2 – Day4	0.460
Day2 – Day5	0.993
Day2 – Day6	0.436
Day3 – Day4	0.505
Day3 – Day5	0.144
Day3 – Day6	0.522
Day4 – Day5	0.334
Day4 – Day6	0.135
Day5 – Day6	0.634

Table S4: Statistical results (for Figure 3) on the effect of cross-pollination, geitonogamous pollination and autonomous self-pollination on relative seed set (Kruskal-Wallis test). Significant results are indicated in bold.

Flower colour: red

Trait	Factor	**p-value**	**Chi²**	**df**
**Relative seed set**	**Pollination treatment**	**<0.001**	23.23	2

Dunn's post-hoc (Bonferroni)	**p-value**
cross– geito	0.214
cross – self	**<0.001**
geito – self	**0.007**

Flower colour: purple

Trait	Factor	**p-value**	**Chi²**	**df**
**Relative seed set**	**Pollination treatment**	**<0.001**	18.97	2

Dunn's post-hoc (Bonferroni)	**p-value**
cross – geito	0.113
cross – self	**<0.001**
geito – self	0.104

Table S5: Statistical results (for Figure 4) on the effect of flower colour of pollen donor and pollen receiver on relative seed set (Kruskal-Wallis test). Significant results are indicated in bold.

Trait	Factor	**p-value**	**Chi²**	**df**
**Relative seed set**	**Flower colour**	0.284	3.79	3

Table S6: Statistical results (for Figure 6) on the effect of treatment (covered, free, hand + free), flower colour (red, purple) and population type (monomorphic, polymorphic) on relative seed set (mixed effect model; quasibinomial). Post-hoc results are grouped by treatment (A) and population type (B). Significant results are indicated in bold.

Trait	Factor	**p-value**	**Chi²**	**df**
**Relative seed set**	**Treatment**	**<0.001**	181.84	2
	**Flower colour**	**<0.001**	12.41	1
	**Population**	0.132	2.27	1
	**Treatment : Flower colour**	0.072	5.27	2
	**Treatment : Population**	0.098	4.65	2
	**Flower colour : Population**	**0.019**	5.46	1
	**Treatment : Flower colour : Population**	0.070	5.31	2

**(A)** Post-hoc ("emmeans")	**p-value**
**Treatment:** covered	
purple (poly) – red (poly)	0.592
purple (poly) – purple (mono)	0.672
purple (poly) – red (mono)	0.999
red (poly) – purple (mono)	0.983
red (poly) – red (mono)	0.635
purple (mono)– red (mono)	0.690

**Treatment:** free	
purple (poly) – red (poly)	**<0.001**
purple (poly) – purple (mono)	0.997
purple (poly) – red (mono)	0.999
red (poly) – purple (mono)	**0.035**
red (poly) – red (mono)	**0.030**
purple (mono)– red (mono)	0.999
**Treatment:** hand + free	
purple (poly) – red (poly)	0.252
purple (poly) – purple (mono)	0.999
purple (poly) – red (mono)	0.994
red (poly) – purple (mono)	0.796
red (poly) – red (mono)	0.561
purple (mono)– red (mono)	0.986

**(B)** Post-hoc ("emmeans")	**p-value**
**Population type:** monomorphic	
red (covered) – red (free)	**<0.001**
red (covered) – red (hand)	**<0.001**
red (hand) – red (free)	**0.001**
purple (covered) – purple (free)	**0.025**
purple (covered) – purple (hand)	**0.002**
purple (hand) – purple (free)	**0.006**
**Population type:** polymorphic	
red (covered) – red (free)	0.589
red (covered) – red (hand)	**0.048**
red (hand) – red (free)	**<0.001**
purple (covered) – purple (free)	**0.001**
purple (covered) – purple (hand)	**<0.001**
purple (hand) – purple (free)	**0.001**

Table S7: Statistical results (for Figure 7A) on the effect of population type (monomorphic, polymorphic), flower colour (red, purple) and year (2022, 2023) on relative seed set (Generalized Linear Mixed Model; quasibinomial). Post-hoc results grouped by population type. Significant results are indicated in bold.

Trait	Factor	p-value	Chi²	df
**Relative seed set**	**Population**	**<0.001**	14.93	2
	**Flower colour**	**<0.001**	30.62	2
	**Year**	0.051	3.82	1
	**Population : Flower colour**	0.769	11.51	1
	**Population : Year**	0.644	0.09	1
	**Flower colour : Year**	**<0.001**	12.67	1
	**Population : Flower colour : Year**	0.291	0.59	1

Post-hoc (“emmeans”)	p-value
**Population:** monomorphic	
red 2022 – red 2023	0.786
purple 2022 – purple 2023	0.141
red 2022 – purple 2022	0.889
red 2023 – purple 2023	0.120
red 2022 – purple 2023	0.501
purple 2022 – red 2023	0.999
**Population:** polymorphic	
red 2022 – red 2023	0.809
purple 2022 – purple 2023	**0.004**
red 2022 – purple 2022	**0.021**
red 2023 – purple 2023	**<0.001**
red 2022 – purple 2023	**<0.001**
purple 2022 – red 2023	**0.013**

Table S8: Statistical results (for Figure 7B) on the effect of population type (monomorphic, polymorphic), flower colour (red, purple) and year (2022, 2023) on number of carpels (Generalized Linear Mixed Model; quasibinomial). Post-hoc results grouped by population type. Significant results are indicated in bold.

Trait	Factor	p-value	Chi²	df
**Number of carpels**	**Population**	**<0.001**	19.74	2
	**Flower colour**	**<0.001**	17.15	2
	**Year**	**<0.001**	19.03	1
	**Population : Flower colour**	**<0.001**	16.24	1
	**Population : Year**	**0.002**	9.33	1
	**Flower colour : Year**	0.809	0.06	1
	**Population : Flower colour : Year**	0.972	0.00	1

Post-hoc ("emmeans")	p-value
**Population:** monomorphic	
red 2022 – red 2023	0.968
purple 2022 – purple 2023	0.927
red 2022 – purple 2022	**0.009**
red 2023 – purple 2023	**0.007**
red 2022 – purple 2023	**0.006**
purple 2022 – red 2023	**0.012**
**Population:** polymorphic	
red 2022 – red 2023	**0.012**
purple 2022 – purple 2023	**<0.001**
red 2022 – purple 2022	0.982
red 2023 – purple 2023	0.999
red 2022 – purple 2023	**0.002**
purple 2022 – red 2023	**<0.001**

Table S9: Statistical results on the relationship between elevation and temperature (Figure 5), flowering peak (Figure 5), pollen limitation PL (Figure 8A), pollinator dependency PD (Figure 8B), pollinator contribution PC (Figure 8C), and pollinator distribution (Figure 9). Generalized linear models; relative seed set: family = quasibinomial; number of carpels, flowering peak, temperature, PL, PD, PC, pollinators: family = gaussian). Significant results are indicated in bold.

Trait	Factor	R^2	p-value	df
**Flowering peak** (Figure 5)	**2023**			
	Elevation (red flowers)	0.73	**0.007**	6
	Elevation (purple flowers)	0.00	0.172	5
**Temperature** (Figure 5)	**2022-2023**			
	Elevation	0.39	**0.013**	13
**Pollen limitation** (Figure 8A)	Elevation (red flowers)	0.62	**0.020**	6
	Elevation (purple flowers)	0.00	0.992	5
**Pollinator dependency** (Figure 8B)	Elevation (red flowers)	0.44	0.072	6
	Elevation (purple flowers)	0.08	0.522	5
**Pollinator contribution** (Figure 8C)	Elevation (red flowers)	0.61	**0.024**	6
	Elevation (purple flowers)	0.26	0.239	5
**Pollinators** (Figure 9)	Beetles	0.32	**0.022**	15
	Bees	0.25	0.051	15

Table S10: Statistical results (Appendix S1, Figure S4) on the effect of seasonal phase (early, mid, late), flower colour (red, purple) and population type (monomorphic, polymorphic) on relative seed set (mixed effect model; quasibinomial). Significant results are indicated in bold.

Trait	Factor	p-value	Chi²	df
**Relative seed set**	**Season**	**<0.001**	38.65	2
	**Flower colour**	**<0.001**	53.26	1
	**Population**	0.299	1.08	1
	**Season : Flower colour**	**0.001**	13.41	2
	**Season : Population**	0.644	0.88	2
	**Flower colour : Population**	**<0.001**	17.71	1
	**Season : Flower colour : Population**	**0.048**	6.04	2

Post-hoc ("emmeans")	p-value
**Population:** red monomorphic	
Early – late	**0.007**
Early – mid	**0.039**
Late – mid	0.651
**Population:** polymorphic	
**red**	
Early – late	**0.042**
Early – mid	**0.026**
Late – mid	0.960
**purple**	
Early – late	0.516
Early – mid	**0.002**
Late – mid	**0.034**
**Population:** purple monomorphic	
Early – late	0.998
Early – mid	**<0.001**
Late – mid	**<0.001**

Table S11: Statistical results (Figure 5) on the effect of population type (monomorphic, polymorphic) and flower colour (red, purple) on flowering peak (linear mixed model; gaussian). Significant results are indicated in bold

Trait	Factor	**p-value**	**Chi²**	**df**
**Flowering peak**	**Population type**	**0.034**	4.50	1
	**Flower colour**	**<0.001**	30.49	1
	**Population type : Flower colour**	**<0.001**	15.43	1

Post-hoc (“emmeans”)	**p-value**
**Flowering peak**	
purple (poly) – red (poly)	**0.018**
purple (poly) – purple (mono)	0.814
purple (poly) – red (mono)	0.993
red (poly) – purple (mono)	0.056
red (poly) – red (mono)	**0.014**
purple (mono)– red (mono)	0.920

Table S12: Statistical results (Figure 9) on (**A**) the effect of elevation and pollinator group (bees, beetles) on pollinator occurrence (generalized linear model; gaussian); the effect of trap colour (red, purple, blue, white), population type (monomorphic red, monomorphic purple, polymorphic) on number of individuals caught (generalized linear model; gaussian) for beetles (**B**) and bees (**C**). Significant results are indicated in bold.

A

Trait	Factor	**p-value**	**Chi²**	**df**
**Individuals per hour and trap**	**Elevation**	**0.004**	9.37	1
	**Pollinator group**	0.397	0.72	1
	**Elevation : Pollinator group**	0.052	3.78	1

B

Trait	Factor	**p-value**	**Chi²**	**df**
**Number of beetles**	**Trap colour**	**<0.001**	38.19	3
	**Population type**	0.196	3.26	2
	**Trap colour : Population type**	0.182	8.72	6

Post-hoc ("emmeans")	**p-value**
blue – purple	0.992
blue – red	**<0.001**
blue – white	1.000
purple – red	**<0.001**
purple – white	0.993
red – white	**<0.001**

C

Trait	Factor	**p-value**	**Chi²**	**df**
**Number of bees**	**Trap colour**	**<0.001**	20.84	3
	**Population type**	0.880	0.254	2
	**Trap colour : Population type**	0.444	5.817	6

Post-hoc ("emmeans")	**p-value**
blue – purple	0.192
blue – red	0.518
blue – white	0.121
purple – red	**0.007**
purple – white	0.995
red – white	**0.004**
